# Impact of frailty on early rhythm control outcomes in older adults with atrial fibrillation: A nationwide cohort study

**DOI:** 10.3389/fcvm.2022.1050744

**Published:** 2023-01-06

**Authors:** Ga-In Yu, Daehoon Kim, Jung-Hoon Sung, Eunsun Jang, Hee Tae Yu, Tae-Hoon Kim, Hui-Nam Pak, Moon-Hyoung Lee, Gregory Y. H. Lip, Pil-Sung Yang, Boyoung Joung

**Affiliations:** ^1^Division of Cardiology, Department of Internal Medicine, Yonsei University College of Medicine, Seoul, Republic of Korea; ^2^Division of Cardiology, CHA Bundang Medical Center, CHA University, Seongnam, Republic of Korea; ^3^Liverpool Centre for Cardiovascular Science, University of Liverpool and Liverpool Heart & Chest Hospital, Liverpool, United Kingdom

**Keywords:** atrial fibrillation, rhythm-control, rate-control, frailty, older adults

## Abstract

**Purpose:**

Rhythm-control therapy administered early following the initial diagnosis of atrial fibrillation (AF) has superior cardiovascular outcomes compared to rate-control therapy. Frailty is a key factor in identifying older patients’ potential for improvement after rhythm-control therapy. This study evaluated whether frailty affects the outcome of early rhythm-control therapy in older patients with AF.

**Methods:**

From the Korean National Health Insurance Service database (2005–2015), we collected 20,611 populations aged ≥65 years undergoing rhythm- or rate-control therapy initiated within 1 year of AF diagnosis. Participants were emulated by the EAST-AFNET4 trial, and stratified into non-frail, moderately frail, and highly frail groups based on the hospital frailty risk score (HFRS). A composite outcome of cardiovascular-related mortality, myocardial infarction, hospitalization for heart failure, and ischemic stroke was compared between rhythm- and rate-control.

**Results:**

Early rhythm-control strategy showed a 14% lower risk of the primary composite outcome in the non-frail group [weighted incidence 7.3 vs. 8.6 per 100 person-years; hazard ratio (HR) 0.86, 95% confidence interval (CI) 0.79–0.93, *p* < 0.001] than rate-control strategy. A consistent trend toward a lower risk of early rhythm-control was observed in the moderately frail (HR 0.91, 95% CI 0.81–1.02, *p* = 0.09) and highly frail (HR 0.93, 95% CI 0.75–1.17, *p* = 0.55) groups.

**Conclusion:**

Although the degree attenuated with increasing frailty, the superiority of cardiovascular outcomes of early rhythm-control in AF treatment was maintained without increased risk for safety outcomes. An individualized approach is required on the benefits of early rhythm-control therapy in older patients with AF, regardless of their frailty status.

## Introduction

Atrial fibrillation (AF) has the highest proportion among persistent arrhythmias, and its prevalence increases with aging (1, 2). It can be related with ischemic stroke, hospitalization, heart failure (HF), as well as cognitive dysfunction, depression, and impaired quality of life. It ultimately increases mortality (3, 4). Many major clinical studies have been conducted to compare rate-control and rhythm-control treatment strategy in AF treatment (5–8). The results have shown the superiority of rhythm-control, strengthened by recent studies on the development of newer medications and advances in ablation capable of overcoming the limitations of the initial rhythm-control strategies (9). Additionally, it has been shown that these reference trial results are equally reflected in real world observational data (10). However, the outcome of rhythm-control for older patients is still controversial. In an analysis of the AFFIRM trial for ages between 70 and 80 years, rate-control therapy had lower mortality and hospitalization rates than rhythm-control therapy (11). However, a study showed that active rhythm-control with ablation is advantageous in the older population (12).

Frailty refers to a condition in which the physiological system that copes with external stress weakens and becomes functionally vulnerable with increasing age. It has a significant impact on medical outcomes of the older population (13), and has been found to be an important factor in predicting older patients’ potential for improvement after catheter ablation (14, 15). Therefore, the assessment of frailty plays a meaningful role in generating management plans for older patients (16). The method of measuring frailty is systematic and sufficiently objective, and validation has already been made through the results of studies on older population in various countries (17–19).

The results of studies on rhythm-control in compared to rate-control the older AF population have been mostly associated with age. However, the effects of variables apart from age have been insufficiently studied. In this study, the effect of frailty on the results of early rhythm-control compared to rate-control therapy in the older AF population was evaluated.

## Materials and methods

The present study is a retrospective observational cohort analyses based on the National Health Claims Database (NHIS-2016-4-009) provided by the National Health Insurance Service (NHIS) of Republic of Korea. The start of the observation period was 1 January 2005. The NHIS is the single insurer managed by the Korean Government, with the majority (97.1%) of Korean citizens as mandatory subscribers, and the remaining (3%) under the Medical Aid program. As the NHIS database contains the information of Medical Aid users as well, it is essentially based on the entire Korean population (4, 20–23). The data can be accessed through the National Health Insurance Data Sharing Service homepage.^1^

This study was approved by the Institutional Review Board of the Yonsei University Health System (4-2016-0179), and following strict confidentiality guidelines, personally identifiable information was removed after the cohort was created, and it was therefore exempt from prior consent requirements. Applications to use the NHIS data will be reviewed by the inquiry committee of research support and, once approved, raw data will be provided to the authorized researcher with a fee at several permitted sites. Through this study, we attempted to closely emulate the protocol of the EAST-AFNET4 trial, as summarized in Table 1.

**TABLE 1:** Summary of strategies for emulating target trial.

Components	Target trial (EAST-AFNET4)	This study
Inclusion period	28 July 2011 – 30 December 2016	1 January 2005 – 31 December 2015
Eligibility criteria	1) Adults (≥18 years of age) who were older than 75 years of age, had had a previous transient ischemic attack or stroke, or met two of the following criteria: age greater than 65 years, female sex, heart failure, hypertension, diabetes mellitus, severe coronary artery disease, chronic kidney disease, and left ventricular hypertrophy 2) Early AF (diagnosed ≤12 months before enrollment)	1) Selected older adults (≥65 years of age) that received a rhythm-control or rate-control treatments and have no prior history of prescriptions and no records of ablation in the database who were older than 75 years of age, had a previous transient ischemic attack or stroke, or met two of the following criteria: age greater than 65 years, female sex, heart failure, hypertension, diabetes mellitus, myocardial infarction, and chronic kidney disease 2) Early AF (defined as AF diagnosed ≤12 months before enrollment) 3) Undergoing oral anticoagulation (>90 days of supply within 180 days after their first recorded prescription of rhythm- or rate-control medications or ablation procedure)
Exposed group	Rhythm control: antiarrhythmic drugs, AF ablation, cardioversion of persistent AF, to be initiated early after randomization	Rhythm control: a prescription of more than a 90-day supply of any antiarrhythmic drugs in the 180-day period since the first prescription or the performance of an ablation procedure for AF.
Unexposed group	Usual care: initially treated with rate-control therapy without rhythm-control therapy	Rate control: a prescription of more than a 90-day supply of any rate-control drugs in the 180-day period since the first prescription and with no prescription of rhythm-control drug and no ablation within this period. Patients prescribed rhythm-control drugs for more than 90 days or who underwent ablation within the 180-day period since the initiation of rate-control drugs were classified as intention-to-treat with rhythm control.
Primary outcome	1) A composite of death from cardiovascular causes, stroke, or hospitalization with worsening of heart failure or acute coronary syndrome 2) The number of nights spent in the hospital per year.	1) A composite of death from cardiovascular causes, ischemic stroke, hospitalization for heart failure, or acute myocardial infarction 2) The number of nights spent in the hospital per year.
Secondary outcome	Each component of the primary outcome, rhythm, left ventricular function, quality of life, AF-related symptom	Each component of the primary outcome
Safety outcome	A composite of death from any cause, stroke, or pre-specified serious adverse events of special interest capturing complications of rhythm-control therapy	A composite of death from any cause, intracranial or gastrointestinal bleeding requiring hospitalization, or pre-specified serious adverse events of special interest capturing complications of rhythm control
Follow-up	From randomization until the end of the trial, death, or withdrawal from the trial.	From 180 days after their first recorded prescription or procedure to avoid immortal time bias until the end of follow-up of the database (31 December 2016) or death.

### Study population

This observational cohort study evaluated whether the degree of frailty affects the outcome of rhythm- and rate-control therapies in older AF populations. AF was defined based on the cases registered with the National Health Claims Database as International Classification of Diseases 10th Revised Edition (ICD-10) code I48, and the time of initial diagnosis was judged to be the time when the code was first registered. The code I48 registration was only possible when AF was documented in electrocardiogram (ECG). The reliability of AF diagnosis using this method in the NHIS database was verified as a positive predictive value of 94.1% in a previous study (22).

We designed this study based on the criteria of the EAST-AFNET 4 trial, which has been approved for the study of AF early rhythm control (9). We collected AF populations above 65 years of age with a medical history of an ischemic stroke or transient ischemic attack, or ones that met the following standards: female, with the presence of any of the related medical history (hypertensive disorders, diabetes, chronic renal disease, HF, or previous myocardial infarction) between 1 January 2005 and 31 December 2015 (details of inclusion and exclusion standards are described in Table 1).

Patients underwent rhythm-control or rate-control treatments according to a new-user and intention-to-treat design. A “new-user” was defined as a patient with no previous record of prescription or treatment during the observation period, while “intention-to-treat-with-rhythm-control” was defined as a patient prescribed with rhythm control drugs for 90 days within 180 days from the first prescription after AF diagnosis, or the first prescription after AF procedure. In the case of patients who underwent ablation, the intention-to-treat-with-rhythm-control group was considered only if the procedure was carried out within 180 days after the initial diagnosis of AF. Conversely, “intention-to-treat-with-rate-control” was defined as having been prescribed a rate-control drug for 90 days within 180 days from the first prescription after AF diagnosis, and not receiving any rhythm-control drug prescriptions or ablation within this period. Definitions and ICD-10 codes used for defining rhythm- and rate- control drugs treatments and procedures for AF are presented in Supplementary Table 1.

**FIGURE 1:**
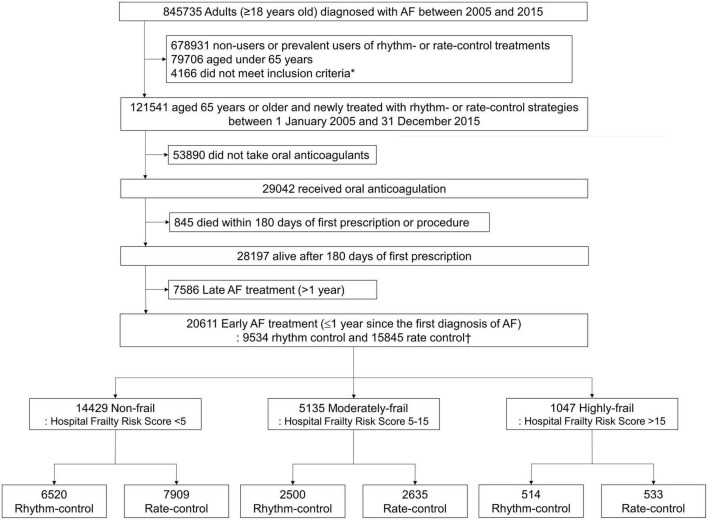
Flowchart of enrollment and analysis of the study population. AF, atrial fibrillation. *AF populations above 65 years of age with a medical history of an ischemic stroke or transient ischemic attack, or ones that met the following standards: female, with the presence of any of the related medical history (hypertensive disorders, diabetes, chronic renal disease, heart failure, or previous myocardial infarction).

The present study excluded the following: (1) patients who had not been prescribed anticoagulants (warfarin or direct oral anti-coagulant) for 90 days or more within 180 days of starting (rhythm-control or rate-control) drugs therapy or receiving ablation for AF, and (2) those who died within 180 days of starting drugs therapy or undergoing ablation (Figure 1).

This study assessed the frailty of individual patients using hospital frailty risk score (HFRS) based on administrative data (18). The HFRS has been validated using data from Canada, the UK, and Korea; therefore, it is a reliable and objective indicator (17, 19, 24). The HFRS for each patient was estimated using ICD-10 codes registered before 180 days from the first prescription date after AF diagnosis. The ICD-10 code-based HFRS is a scoring method based on the selection of 109 diagnostic ICD codes related to frailty, assigning a specific value proportional to how strongly it was reflected for each code (17). The variables and their corresponding ICD codes are described in Supplementary Table 2.

We classified the enrolled populations into three levels of frailty groups according to the calculated HFRSs. Populations were divided into the non-frail (low-risk) (<5), moderately frail (5–15), and highly frail (>15) groups with reference to previously reported cut-off points (17). The interaction tests were performed among three groups.

### Outcomes and covariates

Outcomes and covariates were obtained from the Korea NHIS data. To avoid immortal time bias, the investigation of clinical outcomes was begun 180 days after the first prescription or first ablation post AF diagnosis, and the observation ended (31 December 2016) according to the protocol or with the death of the participant. The types and definitions of AF procedures and the corresponding ICD-10 codes are summarized in Supplementary Table 1 in detail. The endpoint of the study also followed the EAST-AFNET 4 trial with evidence (9).

The primary outcome was a composite of ischemic stroke, acute myocardial infarction, hospitalization for HF, and cardiovascular mortality. Additionally, the number of days in hospital per year during each patient’s individual follow-up period was also identified and calculated. The safety outcome was a composite of all-cause death, major bleeding (intracranial or gastrointestinal bleeding), and critical adverse events associated with rhythm-control (complications related to ablation such as cardiac tamponade, and the development of bradyarrhythmia related to antiarrhythmic drugs). The definition of study outcomes and the ICD codes corresponding to each element are detailed in Supplementary Table 3.

### Statistical analysis

Descriptive statistics were employed to analyze the baseline characteristics of participants. In order to eliminate bias between the rhythm- and rate-control groups, propensity score overlap weighting was performed on the baseline characteristics (25). Propensity scores for the probability of receiving rhythm-control were estimated by logistic regression based on demographics, time since diagnosis of AF, year of treatment initiation, level of care at which the initial prescription was provided, clinical risk scores, medical history, and concurrent medication use (variables in Supplementary Table 4). We estimated the balance between enrolled patients by standardized differences of every qualitative and quantitative covariates using a threshold of 0.1 to manifest imbalance (26). The distribution of propensity scores before and after overlap weighting is shown in Supplementary Figure 1. We did subgroup analyses for the primary composite outcome stratified by sex, age, HF, chronic kidney disease, and ischemic stroke. Interaction tests were done for all subgroups. We used the test variable from the weighting procedure to recreate the overlap weighting.

The weighted incidence rates of clinical outcomes were evaluated by dividing the weighted number of events by 100 person-years at risk, with a 95% confidence interval (CI). The significance of the difference in outcome between the rhythm-control group and the rate-control group was confirmed using the log-rank test, and the results were expressed through failure curves. Fine and Gray competing risk regression with time-varying covariates was used to estimate the relative hazards of all-cause mortality as a main outcome (27). The proportional hazards assumption was checked using the Schoenfeld residual test (28).

Cox models were stratified on frailty score, with treatment as the exposure. Cox proportional hazards model for the total weighted study population was used to evaluate whether the degree of frailty (non-frailty, moderately frailty, or highly frailty) affects the primary composite outcome and safety outcome of the early AF treatment strategy (rhythm-control vs. rate-control). The balance of baseline characteristics before and after propensity overlap weighting in the overall study population is summarized in Supplementary Table 5.

A two-sided *p*-value < 0.05 was judged to be objectively significant. All statistical work was performed with the R version 4.1.2 (The R Foundation^2^, Vienna, Austria) and the SAS version 9.4 (SAS Institute, Cary, NC, USA).

### Sensitivity analyses

Sensitivity analysis was carried out according to the on-treatment principle, by censoring treatments for patients who dropped out mid-therapy or switched strategies between rhythm- and rate-control. Time-varying regression was performed considering a time-dependent variable for the switch between treatments. The above analytical process is schematically represented in Supplementary Figure 2. Next, we performed one-to-one propensity score matching test without replacement with a caliper of 0.01. The balance of baseline characteristics after propensity score matching is summarized in Supplementary Table 6. We evaluated the association between intention-to-treat with rhythm control, which was defined as the performance of a cardioversion for AF as well as the use of antiarrhythmic drug or ablation, and cardiovascular outcomes. We defined the treatment strategies of rhythm or rate control as a prescription for more than a 20-day supply of the drugs in the 30-day period since the first prescription, instead of the 180-day period in the main analyses. Follow-up began 30 days after the first recorded prescription or procedure to avoid immortal time bias. Any systematic bias in the present study was excluded by using falsification analysis with 30 pre-specified falsification endpoints with a true hazard ratio (HR) of 1 (29). The component and their corresponding ICD codes for falsification endpoints are described in Supplementary Table 7.

## Results

**FIGURE 2:**
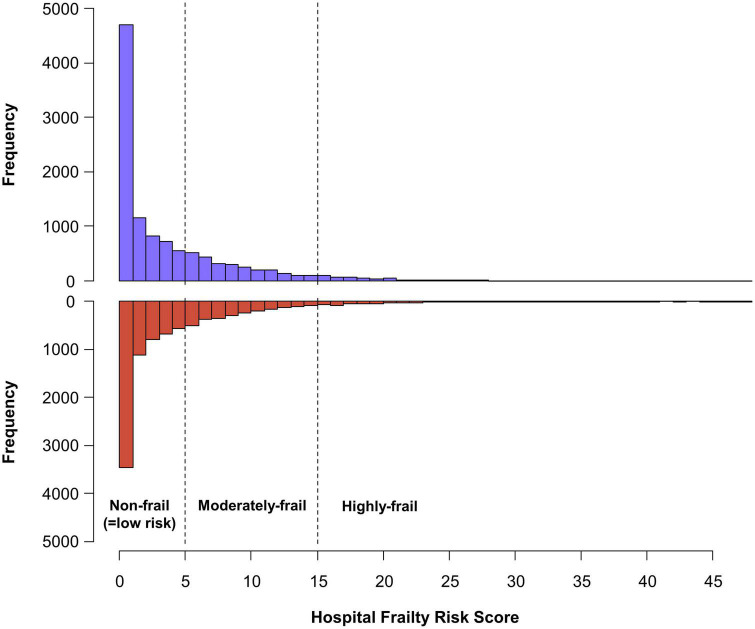
Distribution of the hospital frailty risk score in study population recently diagnosed with atrial fibrillation. The patients were diagnosed within 1 year, receiving new rhythm-control or rate-control treatments among patients.

**FIGURE 3:**
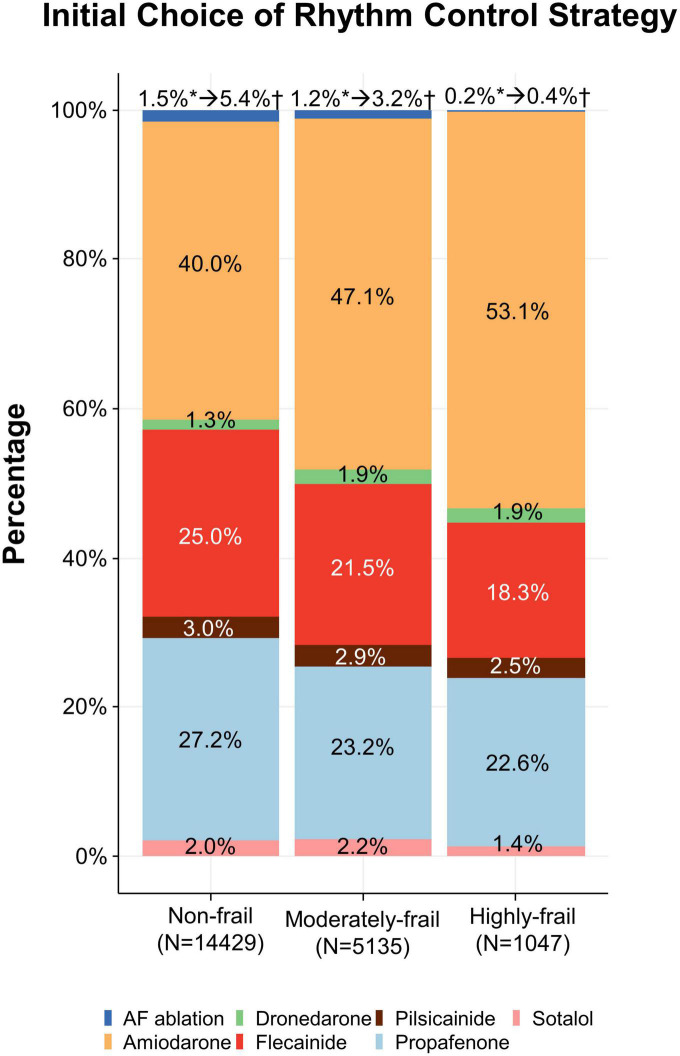
Initial choice of rhythm-control strategy. Treatments according to different frailty risk categories among patients who were recently (within 1 year) diagnosed with atrial fibrillation.

A total of 20,611 patients aged ≥65 years [median 73, interquartile range (IR) 69–78] at an early stage of AF diagnosis (within 1 year) were included (Figure 1). Before propensity overlap weighting, compared with those in the rate-control group, populations in the rhythm-control group were younger, mostly female, had a higher income, and more comorbidities, irrespective of frailty risk (Supplementary Table 4). After propensity overlap weighting, the baseline characteristics were well balanced between the rhythm-control and rate-control groups (Table 2). The small sized hospital showed a preference for rate-control approach independent of frailty status (Supplementary Table 4). The distribution of HFRS in the population recently diagnosed with AF receiving rhythm-control or rate-control strategies is presented in Figure 2. In rhythm-control therapy, class III antiarrhythmic drug, amiodarone, had the highest proportion (40% in the non-frail, 47.1% in the moderately frail, and 53.1% in the highly frail groups), followed by the class Ic antiarrhythmic drugs (Figure 3). Ablation strategy was performed as an initial treatment in 1.5, 1.2, and 0.2% of the patients in the non-frail, moderately frail, and highly frail groups, respectively, and as a final therapy during the entire study period in 5.4, 3.2, and 0.4% of the patients in each group, respectively (Figure 3).

**TABLE 2:** Baseline characteristics before overlap weighting.

	Non-frail (*n* = 14,429)			Moderately frail (*n* = 5,135)			Highly frail (*n* = 1,047)		
Variables	Rhythm control (*n* = 6,520)	Rate control (*n* = 7,909)	ASD (%)	Rhythm control (*n* = 2,500)	Rate control (*n* = 2,635)	ASD (%)	Rhythm control (*n* = 514)	Rate control (*n* = 533)	ASD (%)
Age (years)	72 (68–76)	73 (69–78)	23.9	74 (70–79)	75 (71–80)	18.6	76 (71–81)	77 (72–83)	18.8
65–74	4,349 (66.7)	4,548 (57.5)	19.0	1,314 (52.6)	1,166 (44.3)	16.7	215 (41.8)	194 (36.4)	11.1
≥75	2,171 (33.3)	3,361 (42.5)	19.0	1,186 (47.4)	1,469 (55.7)	16.7	299 (58.2)	339 (63.6)	11.1
Male	1,776 (49.5)	1,827 (51.6)	7.5	1,121 (44.8)	1,247 (47.3)	5.0	187 (36.4)	234 (43.9)	15.4
AF duration (months)	0.1 (0.0–1.2)	0.0 (0.0–0.0)	30.5	0.0 (0.0–1.2)	0.0 (0.0–0.2)	22.9	0.0 (0.0–1.3)	0.0 (0.0–1.0)	1.9
**Enrollment year:**									
2005	353 (5.4)	1,078 (13.6)	28.3	65 (2.6)	146 (5.5)	14.9	7 (1.4)	18 (3.4)	13.3
2006	374 (5.7)	841 (10.6)	17.9	83 (3.3)	157 (6.0)	12.6	8 (1.6)	21 (3.9)	14.6
2007	351 (5.4)	632 (8.0)	10.5	92 (3.7)	146 (5.5)	8.9	13 (2.5)	16 (3.0)	2.9
2008	339 (5.2)	628 (7.9)	11.1	106 (4.2)	193 (7.3)	13.2	13 (2.5)	30 (5.6)	15.7
2009	378 (5.8)	555 (7.0)	5.0	122 (4.9)	133 (5.0)	0.8	22 (4.3)	29 (5.4)	5.4
2010	454 (7.0)	526 (6.7)	1.2	166 (6.6)	189 (7.2)	2.1	29 (5.6)	38 (7.1)	6.1
2011	569 (8.7)	539 (6.8)	7.1	212 (8.5)	230 (8.7)	0.9	42 (8.2)	39 (7.3)	3.2
2012	655 (10.0)	615 (7.8)	8.0	242 (9.7)	261 (9.9)	0.8	55 (10.7)	47 (8.8)	6.3
2013	800 (12.3)	739 (9.3)	9.4	369 (14.8)	320 (12.1)	7.7	78 (15.2)	79 (14.8)	1.0
2014	937 (14.4)	731 (9.2)	15.9	426 (17.0)	348 (13.2)	10.7	112 (21.8)	76 (14.3)	19.7
2015	1,310 (20.1)	1,025 (13.0)	19.3	617 (24.7)	512 (19.4)	12.7	135 (26.3)	140 (26.3)	<0.1
High tertile of income	3,419 (52.4)	3,168 (40.1)	25.0	1,219 (48.8)	1,164 (44.2)	9.2	256 (49.8)	263 (49.3)	0.9
Number of OPD visits ≥12/year	5,813 (89.2)	6,313 (79.8)	26.0	2,174 (87.0)	1,964 (74.5)	31.9	387 (75.3)	346 (64.9)	22.8
Living in metropolitan areas	3,230 (49.5)	3,284 (41.5)	16.2	1,097 (43.9)	994 (37.7)	12.6	203 (39.5)	196 (36.8)	5.6
**Level of care initiating treatment**									
Tertiary	4,019 (61.6)	3,120 (39.4)	45.5	1,348 (53.9)	1,030 (39.1)	30.1	257 (50.0)	176 (33.0)	35.0
Secondary	2,199 (33.7)	3,899 (49.3)	32.0	1,104 (44.2)	1,489 (56.5)	24.9	251 (48.8)	338 (63.4)	29.7
Primary	302 (4.6)	890 (11.3)	24.7	48 (1.9)	116 (4.4)	14.2	6 (1.2)	19 (3.6)	15.8
CHA_2_DS_2_-VASc score	4 (3–5)	4 (3–5)	15.2	5 (4–6)	5 (4–6)	20.7	6 (5–7)	6 (5–7)	33.3
mHAS-BLED score*	3 (2–3)	2 (2–3)	38.8	3 (3–4)	3 (2–4)	34.7	4 (3–4)	3 (3–4)	37.4
Charlson comorbidity index	3 (2–5)	2 (1–4)	53.4	5 (3–7)	4 (2–6)	49.1	7 (5–9)	6 (4–8)	44.9
Hospital frailty risk score	0.8 (0.0–2.5)	0.0 (0.0–2.2)	13.1	8.0 (6.2–10.3)	8.0 (6.2–10.4)	1.2	19.0 (16.8–23.2)	19.0 (16.6–23.1)	3.0
**Medical history**									
Heart failure	2,761 (42.3)	4,049 (51.2)	17.8	1,331 (53.2)	1,276 (48.4)	9.6	315 (61.3)	256 (48.0)	26.9
Previous hospitalization for heart failure	705 (10.8)	1,288 (16.3)	16.0	427 (17.1)	397 (15.1)	5.5	106 (20.6)	65 (12.2)	22.9
Hypertension	5,432 (83.3)	4,945 (62.5)	48.1	2,187 (87.5)	1,821 (69.1)	45.7	476 (92.6)	410 (76.9)	44.7
Diabetes	1,700 (26.1)	1,553 (19.6)	15.4	929 (37.2)	744 (28.2)	19.1	246 (47.9)	199 (37.3)	21.4
Dyslipidaemia	5,234 (80.3)	4,493 (56.8)	52.2	2,183 (87.3)	1,945 (73.8)	34.6	465 (90.5)	449 (84.2)	18.8
Ischemic stroke	1,456 (22.3)	1,678 (21.2)	2.7	1,279 (51.2)	1,478 (56.1)	9.9	385 (74.9)	420 (78.8)	9.2
Transient ischemic attack	577 (8.8)	401 (5.1)	14.9	410 (16.4)	290 (11.0)	15.7	126 (24.5)	87 (16.3)	20.4
Intracranial bleeding	50 (0.8)	66 (0.8)	0.8	94 (3.8)	113 (4.3)	2.7	56 (10.9)	57 (10.7)	0.6
Myocardial infarction	548 (8.4)	442 (5.6)	11.1	383 (15.3)	256 (9.7)	17.0	112 (21.8)	82 (15.4)	16.5
Peripheral arterial disease	962 (14.8)	625 (7.9)	21.7	485 (19.4)	297 (11.3)	22.7	130 (25.3)	91 (17.1)	20.2
Valvular heart disease	517 (7.9)	876 (11.1)	10.7	230 (9.2)	167 (6.3)	10.7	29 (5.6)	21 (3.9)	8.0
Chronic kidney disease	267 (4.1)	202 (2.6)	8.6	265 (10.6)	128 (4.9)	21.6	99 (19.3)	49 (9.2)	29.1
Proteinuria	366 (5.6)	358 (4.5)	5.0	171 (6.8)	124 (4.7)	9.2	33 (6.4)	28 (5.3)	5.0
Hyperthyroidism	756 (11.6)	559 (7.1)	15.6	379 (15.2)	255 (9.7)	16.7	96 (18.7)	52 (9.8)	25.8
Hypothyroidism	799 (12.3)	533 (6.7)	18.9	408 (16.3)	246 (9.3)	21.0	106 (20.6)	68 (12.8)	21.2
Malignancy	1,407 (21.6)	1,243 (15.7)	15.1	797 (31.9)	659 (25.0)	15.3	188 (36.6)	168 (31.5)	10.7
COPD	1,969 (30.2)	2,165 (27.4)	6.2	1,120 (44.8)	952 (36.1)	17.7	269 (52.3)	251 (47.1)	10.5
Chronic liver disease	2,498 (38.3)	2,031 (25.7)	27.3	1,165 (46.6)	972 (36.9)	19.8	255 (49.6)	223 (41.8)	15.6
Hypertrophic cardiomyopathy	165 (2.5)	101 (1.3)	9.2	71 (2.8)	22 (0.8)	15.0	16 (3.1)	4 (0.8)	17.2
Osteoporosis	2,331 (35.8)	2,233 (28.2)	16.2	1,320 (52.8)	1,220 (46.3)	13.0	361 (70.2)	336 (63.0)	15.3
Sleep apnea	25 (0.4)	10 (0.1)	5.1	7 (0.3)	3 (0.1)	3.7	0 (0.0)	3 (0.6)	10.6
**Concurrent drugs^†^**									
Oral anticoagulant	6,520 (100.0)	7,909 (100.0)	<0.1	2,500 (100.0)	2,635 (100.0)	<0.1	514 (100.0)	533 (100.0)	<0.1
Warfarin	5,627 (86.3)	7,320 (92.6)	20.4	2,090 (83.6)	2,334 (88.6)	14.4	421 (81.9)	443 (83.1)	3.2
NOAC	1,142 (17.5)	774 (9.8)	22.7	540 (21.6)	410 (15.6)	15.6	118 (23.0)	112 (21.0)	4.7
Beta blocker	2,866 (44.0)	4,965 (62.8)	38.4	1,100 (44.0)	1,639 (62.2)	37.1	236 (45.9)	318 (59.7)	27.8
Non-DHP CCB	923 (14.2)	1,386 (17.5)	9.2	330 (13.2)	457 (17.3)	11.5	72 (14.0)	103 (19.3)	14.3
Digoxin	601 (9.2)	3,692 (46.7)	91.9	223 (8.9)	990 (37.6)	72.1	54 (10.5)	180 (33.8)	58.4
Aspirin	1,803 (27.7)	2,002 (25.3)	5.3	655 (26.2)	557 (21.1)	11.9	133 (25.9)	97 (18.2)	18.6
P2Y12 inhibitor	630 (9.7)	565 (7.1)	9.1	299 (12.0)	285 (10.8)	3.6	90 (17.5)	68 (12.8)	13.3
Statin	2,621 (40.2)	2,468 (31.2)	18.9	1,182 (47.3)	1,128 (42.8)	9.0	267 (51.9)	249 (46.7)	10.5
DHP-CCB	1,522 (23.3)	962 (12.2)	29.6	585 (23.4)	400 (15.2)	20.9	122 (23.7)	93 (17.4)	15.6
ACEI/ARB	3,720 (57.1)	4,634 (58.6)	3.1	1,341 (53.6)	1,334 (50.6)	6.0	272 (52.9)	235 (44.1)	17.7
Loop/thiazide diuretics	2,792 (42.8)	4,932 (62.4)	39.9	1,140 (45.6)	1,336 (50.7)	10.2	238 (46.3)	214 (40.2)	12.4
K+ sparing diuretics	884 (13.6)	2,164 (27.4)	34.7	382 (15.3)	528 (20.0)	12.5	81 (15.8)	83 (15.6)	0.5
Alpha blocker	194 (3.0)	221 (2.8)	1.1	70 (2.8)	88 (3.3)	3.1	13 (2.5)	19 (3.6)	6.0

Values are presented as median (interquartile range) or n (%).

*Modified HAS-BLED = hypertension, 1 point; >65 years old, 1 point; stroke history, 1 point; bleeding history or predisposition, 1 point; labile international normalized ratio, not assessed; ethanol or drug abuse, 1 point; drug predisposing to bleeding, 1 point.

^†^Defined as a prescription fill of >90 days within 180 days after the first prescription for rhythm- or rate-control drugs or the performance of an ablation procedure for AF.

AAD, antiarrhythmic drug; ACEI, angiotensin converting enzyme inhibitor; AF, atrial fibrillation; ARB, angiotensin II receptor blocker; ASD, absolute standardized difference; CCB, calcium channel blocker; COPD, chronic obstructive pulmonary disease; DHP, dihydropyridine; NOAC, non-vitamin K antagonist oral anticoagulant; OPD, outpatient department.

### Outcomes of early rhythm-control according to different frailty risks

**FIGURE 4:**
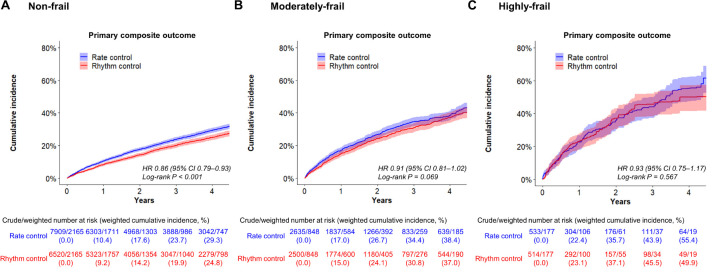
Weighted cumulative incidence curves for primary composite outcome. Curves shown for non-frail **(A)**, moderately frail **(B)**, and highly frail **(C)** patients who were recently (within 1 year) diagnosed with atrial fibrillation.

In non-frail patients (HFRS <5), 6,520 and 7,909 patients started rhythm-control and rate-control therapies at an early stage of AF diagnosis (within 1 year), respectively. During a median follow-up of 4.1 (IR 2.2–7.1) years, early rhythm-control had a lower risk of primary composite outcome compared to rate-control therapy (weighted incidence rate 7.3 vs. 8.6 events per 100 person-years; HR 0.86, 95% CI 0.79–0.93; Table 3 and Figure 4A). For each component of the primary composite outcome, early rhythm-control was related with reduced risks of ischemic stroke (HR 0.75, 95% CI 0.66–0.84), hospitalization for HF (HR 0.86, 95% CI 0.77–0.97) and acute myocardial infarction (HR 0.60, 95% CI 0.43–0.85) when compared to rate-control (Table 3).

**TABLE 3:** Efficacy outcomes in weighted patients undergoing rhythm or rate control stratified according to frailty.

	Rhythm control			Rate control			Absolute rate difference per 100 person-years* (95% CI)	Weighted hazard ratio (95% CI)	*p*-value	*p* for interaction*
Outcome	Number of events	Person-years	Event rate	Number of events	Person-years	Event rate				
**Non-frail (HFRS <5)**	*n* = 2,165.2			*n* = 2,165.2						
Primary composite outcome	581	8,006	7.3	654	7,582	8.6	−1.4 (−2.3 to −0.5)	0.86 (0.79–0.93)	<0.001	0.180
**Components of primary outcome**										
Cardiovascular death	218	9,291	2.3	233	9,085	2.6	−0.2 (−0.7 to 0.2)	0.93 (0.82–1.06)	0.287	0.604
Ischemic stroke	242	8,675	2.8	316	8,332	3.8	−1.0 (−1,5 to −0.5)	0.75 (0.66–0.84)	<0.001	0.245
Hospitalization for heart failure	285	8,526	3.3	326	8,246	4.0	−0.6 (−1.2 to −0.0)	0.86 (0.77–0.97)	0.010	0.168
Acute myocardial infarction	27	9,231	0.3	45	8,976	0.5	−0.2 (−0.4 to −0.0)	0.60 (0.43–0.85)	0.004	0.823
Night spent in hospital/year^†^	17.6 ± 43.5			21.7 ± 52.1			−4.0 (−5.6 to −2.5)		<0.001	
**Moderately frail (HFRS 5–15)**	*n* = 848.2			*n* = 848.2						
Primary composite outcome	270	2,197	12.3	292	2,132	13.7	−1.4 (−3.6 to 0.7)	0.91 (0.81–1.02)	0.093	
**Components of primary outcome**										
Cardiovascular death	118	2,624	4.5	112	2,633	4.3	0.2 (−0.9 to 1.3)	1.06 (0.89–1.27)	0.511	
Ischemic stroke	110	2,406	4.6	136	2,373	5.7	−1.1 (−2.5 to 0.1)	0.80 (0.67–0.95)	0.011	
Hospitalization for heart failure	122	2,386	5.1	132	2,354	5.6	−0.5 (−1.8 to 0.8)	0.92 (0.77–1.09)	0.324	
Acute myocardial infarction	12	2,600	0.5	13	2,602	0.5	−0.0 (−0.4 to 0.3)	0.93 (0.54–1.60)	0.794	
Night spent in hospital/year^†^	46.9 ± 87.5			52.5 ± 92.9			−5.6 (−10.6 to −0.7)		0.025	
**Highly frail (HFRS >15)**	*n* = 177.2			*n* = 177.2						
Primary composite outcome	65	317	20.4	71	326	21.6	−1.2 (−8.3 to 5.9)	0.93 (0.75–1.17)	0.552	
**Components of primary outcome**										
Cardiovascular death	32	391	8.2	35	411	8.4	−0.2 (−4.2 to 3.8)	0.96 (0.69–1.33)	0.866	
Ischemic stroke	27	348	7.7	30	365	8.1	−0.4 (−4.5 to 3.7)	0.93 (0.65–1.33)	0.689	
Hospitalization for heart failure	23	357	6.5	23	376	6.1	0.4 (−3.3 to 4.0)	1.03 (0.69–1.55)	0.873	
Acute myocardial infarction	1	391	0.2	4	398	1.1	−0.8 (−1.9 to 0.3)	0.22 (0.05–1.07)	0.061	
Night spent in hospital/year^†^	110.4 ± 129.7			112.2 ± 131.4			−1.7 (−17.6 to 14.1)		0.829	

Event rates are presented as per 100 person-years. CI, confidence interval; HFRS, hospital frailty risk score.

*p for interactions between frailty risk (non-frail/intermediate-frailty/high-frailty) and treatment strategy (rhythm control or rate control).

^†^Results are reported as mean (standard deviation) and the difference between the treatment groups was estimated using a two-sample weighted t test.

In the moderately frail group (HFRS 5–15), 2,500 and 2,635 patients started rhythm-control and rate-control, respectively, with a median follow-up duration of 2.9 (IR 1.7–5.0) years. In the highly frail group (HFRS >15), rhythm-control and rate-control treatments were started in 514 and 533 patients, respectively, with a median follow-up of 2.1 (IR 1.3–3.7) years. There was no interaction between frailty risk and treatment effect in the primary composite outcome (*p* for interaction = 0.180), any of its components, or the composite safety outcome (*p* for interaction = 0.716). The early rhythm-control strategy showed a non-significant trend toward a lower risk of the primary composite outcome than the rate-control strategy in both the moderately frail (weighted incidence rate 12.3 vs. 13.7 events per 100 person-years; HR 0.91, 95% CI 0.81–1.02; Table 3 and Figure 4B) and highly frail (weighted incidence rate 20.4 vs. 21.6 events per 100 person-years; HR 0.93, 95% CI 0.75–1.17; Table 3 and Figure 4C) groups. Among patients in the moderately frail group, early rhythm-control was related with a reduced risk of ischemic stroke (HR 0.80, 95% CI 0.67–0.95).

We also calculated the number of days in hospital per year during each patient’s individual follow-up period. In both the non-frail and moderately frail groups, the mean number of days in hospital was lower with the early rhythm-control than with the early rate-control group (17.6 vs. 21.7 days per year; *p* < 0.001 and 46.9 vs. 52.5 days per year; *p* = 0.025 respectively; Table 3). In case of the highly frail group, there was no significant difference in the number of days in hospital between the early rhythm-control and early rate-control groups (110.4 vs. 112.2 days per year; *p* = 0.829; Table 3).

In addition, a subgroup analysis according to sex, old age over 75 years, HF, CKD, and ischemic stroke showed that factors other than ischemic stroke in highly frail group did not affect the benefit of the early rhythm control strategy for frail patients (Supplementary Figure 3).

Among all frail groups, there was no significant difference in the risk of composite safety outcome between early rhythm-control and rate-control (Table 4). The weighted incidence rates of early rhythm-control therapy vs. rate-control therapy were 9.0 vs. 9.1 events per 100 person-years (HR 0.99, 95% CI 0.89–1.01, *p* = 0.850); 15.8 vs. 14.1 events per 100 person-years (HR 1.11, 95% CI 0.96–1.30; *p* = 0.149); and 27.3 vs. 27.2 events per 100 person-years (HR 1.00, 95% CI 0.82–1.23; *p* = 0.995) in the non-frail, moderately frail and highly frail groups, respectively (Table 4).

**TABLE 4:** Safety outcomes in weighted patients undergoing rhythm or rate control stratified according to frailty.

	Rhythm control			Rate control			Absolute rate difference per 100 person-years (95% CI)	Weighted hazard ratio (95% CI)	*p*-value
Outcome	Number of events	Person-years	Event rate	Number of events	Person-years	Event rate			
**Non-frail (HFRS <5)**	*n* = 2,165.2			*n* = 2,165.2					
Composite safety outcome*	733	8,110	9.0	746	8,183	9.1	−0.1 (−1.0 to 0.8)	0.99 (0.89–1.01)	0.850
All-cause death	473	9,291	5.1	533	9,085	5.9	−0.8 (−1.5 to −0.1)	0.86 (0.76–0.98)	0.020
Intracranial bleeding	64	9,191	0.7	79	8,938	0.9	−0.2 (−0.5 to 0.1)	0.80 (0.58–1.12)	0.196
Gastrointestinal bleeding	169	8,910	1.9	191	8,694	2.2	−0.3 (−0.7 to 0.1)	0.88 (0.72–1.09)	0.243
**SAE related to rhythm control**									
Cardiac tamponade	7	9,282	0.1	3	9,074	0.0	0.0 (−0.0 to 0.1)	2.09 (0.56–7.84)	0.273
Syncope	129	8,866	1.5	93	8,795	1.1	0.4 (0.0 to 0.7)	1.38 (1.06–1.80)	0.018
Sick sinus syndrome	90	8,783	1.0	26	8,973	0.3	0.7 (0.5 to 1.0)	3.56 (2.30–5.49)	<0.001
Atrioventricular block	41	9,028	0.5	25	8,931	0.3	0.2 (−0.0 to 0.4)	1.70 (1.03–2.81)	0.037
Pacemaker implantation	48	9,019	0.5	16	9,013	0.2	0.3 (0.2 to 0.5)	3.00 (1.71–5.26)	<0.001
Sudden cardiac arrest	59	9,268	0.6	53	9,067	0.6	0.1 (−0.2 to 0.3)	1.12 (0.77–1.61)	0.565
**Moderately frail (HFRS 5–15)**	*n* = 848.2			*n* = 848.2					
Composite safety outcome*	356	2,251	15.8	335	2,381	14.1	1.7 (−0.5 to 4.0)	1.11 (0.96–1.30)	0.149
All-cause death	260	2,624	9.9	263	2,633	10.0	−0.1 (−1.8 to 1.6)	0.99 (0.84–1.18)	0.928
Intracranial bleeding	27	2,582	1.1	25	2,598	1.0	0.1 (−0.4 to 0.6)	1.12 (0.65–1.93)	0.684
Gastrointestinal bleeding	80	2,495	3.2	84	2,497	3.4	−0.2 (−1.2 to 0.9)	0.96 (0.70–1.30)	0.780
**SAE related to rhythm control**									
Cardiac tamponade	2	2,621	0.1	0	2,632	0.0	0.1 (−0.0 to 0.2)	22.5 (2.76–182.7)	0.004
Syncope	50	2,481	2.0	34	2,552	1.3	0.7 (−0.0 to 1.4)	1.52 (0.98–2.36)	0.060
Sick sinus syndrome	25	2,428	1.0	11	2,582	0.4	0.6 (0.1 to 1.1)	2.43 (1.19–4.99)	0.015
Atrioventricular block	16	2,536	0.6	5	2,588	0.2	0.4 (0.1 to 0.8)	3.12 (1.17–8.35)	0.024
Pacemaker implantation	11	2,502	0.4	7	2,600	0.3	0.2 (−0.2 to 0.5)	1.57 (0.61–4.03)	0.352
Sudden cardiac arrest	17	2,619	0.7	25	2,626	1.0	−0.3 (−0.8 to 0.2)	0.70 (0.38–1.28)	0.247
**Highly frail (HFRS >15)**	*n* = 177.2			*n* = 177.2					
Composite safety outcome*	91	335	27.3	96	351	27.2	0.1 (−7.7 to 8.0)	1.00 (0.82–1.23)	0.995
All-cause death	74	391	18.8	78	411	19.1	−0.3 (−6.3 to 5.8)	0.98 (0.78–1.22)	0.842
Intracranial bleeding	9	382	2.4	6	405	1.5	0.9 (−1.1 to 2.8)	1.55 (0.73–3.25)	0.252
Gastrointestinal bleeding	22	364	5.9	24	376	6.5	−0.6 (−4.1 to 3.0)	0.89 (0.59–1.35)	0.597
**SAE related to rhythm control**									
Cardiac tamponade	1	391	0.2	0	411	0.1	0.2 (−0.4 to 0.7)	3.21 (0.22–47.4)	0.395
Syncope	5	380	1.2	10	390	2.6	−1.4 (−3.4 to 5.4)	0.45 (0.20–0.99)	0.048
Sick sinus syndrome	5	371	1.4	0	410	0.1	1.3 (0.1 to 2.6)	13.4 (2.45–73.1)	0.003
Atrioventricular block	2	380	0.6	1	407	0.2	0.4 (−0.5 to 1.3)	2.64 (0.64–11.0)	0.182
Pacemaker implantation	1	381	0.3	0	409	0.1	0.3 (−0.4 to 0.9)	5.44 (0.59–50.2)	0.135
Sudden cardiac arrest	5	385	1.3	8	408	1.9	−0.5 (−2.3 to 1.2)	0.68 (0.32–1.47)	0.503

Event rates are presented as per 100 person-years. CI, confidence interval; SAE, serious adverse event(s).

*p for interactions between frailty risk (non-frail/intermediate frailty/high frailty) and treatment strategy (rhythm control or rate control) was 0.716.

### Sensitivity analysis

Some patients switched between treatment strategies: 1,003 (9.1%) patients from rate control switched to rhythm control, whereas 5,013 (52.6%) patients switched from rhythm control to rate control during follow-up (Supplementary Table 8). The results of on-treatment analyses (Supplementary Table 9) and time-varying regression analyses (Supplementary Table 10) were consistent with the main results. Similar outcomes were obtained in the one-to-one propensity score matched patients as in the main analysis (Supplementary Table 11). The sensitivity analyses in which cardioversion was also included as a rhythm-control strategy, and the results were consistent (Supplementary Table 12). When analyzed using a 30-day observational period (within the period, more than 20 days of drug supply was defined as intention-to-treat), and the results were consistent with the main findings (shown in Supplementary Table 13). In the falsification analysis, the 95% CIs of the correlations between rhythm-control and each falsification endpoint (30 in total) covered 1 of 29 (96.7%), 1 of 29 (96.7%), and 1 of 30 (100%) endpoints in the non-frail, moderately frail, and highly frail groups, respectively (Supplementary Table 14).

## Discussion

Our previous study demonstrated that early rhythm control was associated with less frequent cardiovascular events than rate control in patients with AF (10). In the present study, we conducted a stratified analysis according to frailty, and the main finding were that, compared to early rate-control treatment, early rhythm-control treatment among non-frail patients with AF was related to a 14% decreased risk (absolute decrease in risk: 1.4 events per 100 person-years) in primary efficacy composite outcomes without an increased risk of safety outcomes. These results are consistent with the EAST-AFNET 4 trial that we emulated.

Further, although statistical significance was decreased, a consistent trend toward a lower risk of early rhythm-control was seen in the moderately frail (HR 0.91, 95% CI 0.81–1.02) and highly frail (HR 0.93, 95% CI 0.75–1.17) groups.

Third, there was no difference in the risk of composite safety outcomes across the different frailty groups, which is noteworthy for this study due to the concern that frailty may affect the safety outcome of active rhythm-control therapy.

### Efficacy of early rhythm-control strategy in frail patients

Although current AF guidelines recommend anticoagulation and treatment for comorbidities in all patients who are eligible, rhythm-control treatment is limited to only those who have related symptoms (1, 2). However, the ATHENA and EAST-AFNET 4 trials reported that rhythm-control strategy may reduce cardiovascular events in patients who have received dronedarone (ATHENA) or early rhythm-control therapy (EAST-AFNET 4) (9, 30). We have confirmed through previous works that the results of a reference trial on the benefits of early rhythm-control are equally reflected in real world observational data in old age (10). Nevertheless, it is not yet clear which patients should be targeted for early rhythm-control, especially in elderly patients.

This study is meaningful as it suggests rhythm-control as a suitable target by stratifying the patients according to frailty and extending the inferences. We showed that early rhythm-control strategy was related with a reduced risk of primary outcomes in non-frail patients with AF and there was a consistent trend toward a lower risk of early rhythm-control in the moderate as well as highly frail groups. Thus, early rhythm-control can be carried out without hesitation regardless of the degree of frailty.

### Safety outcomes after early rhythm-control strategy in frail patients

Major guidelines have no specific recommendations on age or frailty assessment for choosing rate- or rhythm-control treatment, such as electrical shock delivery and ablation therapy (1, 2). For safety concerns, rhythm-control therapy is not an active treatment in elderly frail patients; based on the findings of this study, it tends to be a more passive treatment option in primary or secondary institutions. Contrary to common perception however, the results of the present study consistently showed that the degree of frailty had no effect on the safety outcomes of early rhythm-control strategy in older AF populations.

Although the importance of integrated AF management, including symptom control, is consistent in frail patients, the outcome of rhythm-control at an advanced age cannot be guaranteed as frail older patients are predisposed to a decline in both renal and hepatic function, leading to hesitation in its use (19). However, this study showed that early rhythm-control did not affect safety outcomes in older frail AF populations, therefore suggesting that the treatment direction should be decided by evaluating, characterizing, and individualizing each patient’s condition rather than basing it simply on age and frailty. This is also emphasized in the recently revised 2019 AHA/ACC/HRS and 2020 ESC guidelines (1, 2).

### Study limitations

The present research has some limitations, however. First, this study was retrospectively performed using all patients with AF in Korea National Health Insurance Service databases, and needed more validation in general population group. Second, this study used ICD-10 codes for the AF diagnosis, medications and procedure complications. Although AF definition and study outcomes are validated (Supplementary Table 3), there is the possibility of mis-diagnosis of AF and AF ablation state. Third, the number of participants who underwent ablation among patients who chose early rhythm-control as a treatment strategy was low. This is because the reimbursement of ablation therapy is allowed only for AF patients who have not achieved sinus rhythm even after receiving drug treatment, including antiarrhythmic drugs, for 6 weeks or more. The use of catheter ablation therapy was minimal, such that no conclusions could be drawn for this specific form of rhythm-control therapy. Fourth, this study used per-protocol analysis, so there may be an attrition bias resulting from patients who do not have similar characteristics among the groups. Fifth, although the results of the falsification analysis showed that the probability of a major systematic bias was unlikely, unmeasured confounders (such as the adequacy of anticoagulant treatment or health-related habits like drinking, smoking, and physical activity) may have affected the results. Finally, because the aim of the present study was to evaluate the effectiveness of therapeutic interventions for rhythm-control and rate-control, non-users were excluded. However, as some non-users included frail patients that did not need rate-control drugs due to a low baseline heart rate, future studies may need to take this into consideration.

## Conclusion

In the non-frail population, the superiority of cardiovascular outcomes of early rhythm-control in the treatment of AF was observed without any effect on the safety outcomes, showing a consistent trend toward a lower risk of adverse cardiovascular outcomes without an increased risk of safety outcomes. And frailty does not have a detrimental effect on rhythm-control treatment. Thus, an individualized approach is required on the early rhythm-control strategy in older patients with AF, regardless of their frailty status.

## Data availability statement

The datasets presented in this study can be found in online repositories. The names of the repository/repositories and accession number(s) can be found in the article/Supplementary material.

## Ethics statement

This study was approved by the Institutional Review Board of the Yonsei University Health System (4-2016-0179), and following strict confidentiality guidelines, personally identifiable information was removed after the cohort was created, and it was therefore exempt from prior consent requirements. Written informed consent for participation was not required for this study in accordance with the national legislation and the institutional requirements.

## Author contributions

G-IY and DK conceived and designed the study. EJ analyzed the clinical data and drafted the manuscript. HY, T-HK, H-NP, J-HS, and M-HL assisted with data analysis. BJ, P-SY, and GL reviewed the study and put forward constructive suggestions. All authors gave final approval of the version to be published and agreed to be accountable for all aspects of the work.

## Footnotes

1
http://nhiss.nhis.or.kr

2
www.R-project.org
