# Supplementary material for: Impact of frailty on early rhythm control outcomes in older adults with atrial fibrillation: A nationwide cohort study
Source: Front Cardiovasc Med. 2023 Jan 6;9:1050744. doi: 10.3389/fcvm.2022.1050744 (PMC9853018; doi:10.3389/fcvm.2022.1050744)
Supplement: Supplementary file 1 [file Data_Sheet_1.docx]

**SUPPLEMENTAL MATERIAL**

***The impact of frailty on early rhythm control outcome in the elderly population with atrial fibrillation***

| **Contents** | **Page** |
| --- | --- |
| **Supplemental Methods** | 3 |
| **Supplemental Table S1.** Definitions and codes used for defining medical conditions, comorbidities, and drug treatments and procedures for atrial fibrillation. | 4-5 |
| **Supplemental Table S2.** List of 109 variables contributing to calculating the Hospital Frailty Risk Score. | 6-8 |
| **Supplemental Table S3.** Definitions and codes used for study outcomes. | 9 |
| **Supplemental Table S4.** Baseline characteristics after overlap weighting. | 10-11 |
| **Supplemental Table S5.** Baseline characteristics of overall patients undergoing rhythm- and rate-control treatments before and after propensity overlap weighting | 12-13 |
| **Supplemental Table S6.** Baseline characteristics after propensity score matching. | 14-15 |
| **Supplemental Table S7.** Definitions of 35 falsification endpoints. | 16 |
| **Supplemental Table S8.** Crossovers during the follow-up. | 17 |
| **Supplemental Table S9.** Outcomes in weighted patients undergoing rhythm or rate control in on-treatment analyses in which patients were censored patients were censored at the time of crossover between treatment modalities or discontinuation of treatment. | 18 |
| **Supplemental Table S10.** Outcomes in weighted patients undergoing rhythm or rate control in time-varying regression analyses which treatment was treated as a time-dependent variable. | 19 |
| **Supplemental Table S11.** Outcomes in propensity score matched patients undergoing rhythm or rate control. | 20 |
| **Supplemental Table S12.** Outcomes in weighted patients undergoing rhythm or rate control in whom performance of cardioversion within 180 days of their first record of prescription of rate control drugs was treated as intention-to-treat with a rhythm control strategy. | 21 |
| **Supplemental Table S13.** Outcomes in weighted patients undergoing rhythm or rate control defined using a 30-day enrolment period after the first prescription instead of the 180-day period in the main analyses. | 22 |
| **Supplemental Table S14.** Risk of 35 falsification endpoints in weighted patients undergoing rhythm control compared with rate control. | 23 |
| **Supplemental Figure S1.** Distributions of the propensity scores before and after overlap weighting | 24 |
| **Supplemental Figure S2.** Schematic diagram showing the analytical approach used in this study. | 25 |
| **Supplemental Figure S3.** Subgroup analyses of the primary composite outcome. | 26 |

**Supplemental Methods**

***Data Source***

This study is a retrospective analysis based on the national health claims database established by the National Health Insurance Service (NHIS) of Korea. A majority (97.1%) of the Korean population mandatorily subscribes to the NHIS, which is a single insurer managed by the Korean government, with the remaining 3% categorized as medical aid subjects. As the database also includes information of the medical aid population, it can be considered to represent the entire Korean population. All data and materials have been made publicly available at the NHIS. The data can be accessed on the National Health Insurance Data Sharing Service homepage of the NHIS (http://nhiss.nhis.or.kr). Applications to use the NHIS data will be reviewed by the inquiry committee of research support and once approved, raw data will be provided to the authorized researcher with a fee at several permitted sites. This study was approved by the Institutional Review Board of the Yonsei University Health System (4-2016-0179). The requirement for informed consent was waived because personal identification information was removed after cohort generation, in accordance with strict confidentiality guidelines. The NHIS database includes information on drug prescriptions for the entire Korean population from 1 January 2002, which provides a minimum look-back period of 3 years before each person’s date of inclusion (the earliest date of inclusion was 1 January 2005).

***Covariates***

We obtained information regarding selected baseline comorbid conditions for the look-back period from 1 January 2002 up to the start of therapy from inpatient and outpatient hospital diagnoses and pharmacy claims. The patients were considered to have comorbidities when the condition was a discharge diagnosis or was confirmed at least twice in an outpatient setting (Supplemental Table S2). The Hospital Frailty Risk score was calculated retrospectively using 109 ICD-10 diagnostic codes, which were found to be associated with frailty. The baseline relative economic status was determined based on the health insurance premiums in the index year. Concurrent use of medication was verified by identifying NHIS database claims and defined as a prescription of more than a 90-day supply of the medication within the 180 days of the first record of a prescription or procedure for rhythm- or rate-control therapies.

**Supplemental Table S1.** Definitions and codes used for defining medical conditions, comorbidities, and drug treatments and procedures for atrial fibrillation.

|  | **Definitions** | **Codes or conditions** |
| --- | --- | --- |
| **Medical conditions** | |  |
| Atrial fibrillation | Defined from diagnosis* | I48 |
| Heart failure | Defined from diagnosis* | ICD-10: I11.0, I50, I97.1 |
| Heart failure admission history | Defined from principal or first secondary admission diagnoses of heart failure | ICD-10: I11.0, I50, I97.1 |
| Hypertension | Defined if fulfilling both diagnosis* and treatment within 90 days prior to the first recorded prescription or procedure for rhythm or rate control | ICD-10: I10, I11, I12, I13, I15  Treatment: prescription for at least one of all kinds of antihypertensive medication |
| Diabetes mellitus | Defined if fulfilling both diagnosis* and treatment within 90 days prior to the first recorded prescription or procedure for rhythm or rate control | ICD-10: E10, E11, E12, E13, E14  Treatment: prescription for at least one of all kinds of oral antidiabetics or insulin |
| Dyslipidaemia | Defined from diagnosis* | ICD-10: E78 |
| Ischaemic stroke | Defined from diagnosis* | ICD-10: I63, I64 |
| Transient ischaemic attack | Defined from diagnosis* | ICD-10: G45 |
| Intracranial bleeding | Defined from diagnosis* | ICD-10: I60, I61, I62 |
| Myocardial infarction | Defined from diagnosis* | ICD-10: I21, I22, I25.2 |
| Peripheral arterial disease | Defined from diagnosis* | ICD-10: I70.0, I70.1, I70.2, I70.8, I70.9 |
| Valvular heart disease | Defined from diagnoses* mitral stenosis or claims for heart valve surgery | ICD-10: I05.0, I05.2, I34.2, Z95.2-4  Claim for valve replacement or valvuloplasty: O1781, O1782, O1783, O1791, O1792, O1793, O1797, O1794, O1795, O1796, O1798 |
| Chronic kidney disease | Defined from eGFR or diagnosis*  (if laboratory value was not available, diagnosis code was used) | eGFR <60mL/min per 1.73 m^2^  ICD-10: N18, N19 |
| Proteinuria | Defined from laboratory data (if laboratory value was not available, diagnosis code* was used) | Urine dipstick proteinuria 1+ or higher (ICD-10: N06, N391, N392, R80) |
| Hyperthyroidism | Defined from diagnosis* | ICD-10: E05 |
| Hypothyroidism | Defined from diagnosis* | ICD-10: E03 |
| Malignancy | Defined from diagnoses* of cancer (non-benign) | ICD-10: C00-C97 |
| Chronic obstructive pulmonary disease | Defined if fulfilling both diagnosis* and treatment within 90 days prior to the first recorded prescription or procedure for rhythm or rate control | ICD-10: J42, J43(except J43.0), J44  Treatment: SABA, SAMA, LABA, LAMA, ICS, ICS+LABA, or methylxanthine (>1 months). |
| Chronic liver disease | Defined from diagnosis* of chronic liver disease, cirrhosis, and hepatitis | ICD-10: B18, K70, K71, K72, K73, K74, K76.1 |
| Hypertrophic cardiomyopathy | Defined from at least one records of either inpatient or outpatient diagnoses | ICD-10: I42.1, I42.2 |
| Osteoporosis | Defined from diagnosis* | ICD-10: M80, M81, M82 (except M82.0) |
| Sleep apnea | Defined from diagnosis* | ICD-10: G47.3 |
| **Drug treatment for atrial fibrillation (available in South Korea)** | | |
| Anti-arrhythmic drug | |  |
| Class Ic |  | flecainide, pilsicainide, propafenone |
| Class III |  | amiodarone, dronedarone, sotalol |
| Rate control drugs |  |  |
| Beta-blocker |  | atenolol, bisoprolol, carvedilol, metoprol, nebivolol, propranolol, labetalol |
| Calcium channel blocker | | diltiazem, verapamil |
| Cardiac glycosides |  | digoxin |
| **Procedures for atrial fibrillation** | |  |
| Catheter ablation for AF | Defined from admission diagnosis of AF plus claims for ablation procedures | ICD-10: I48  Claim codes: M6542 (Conventional Radiofrequency Ablation of Atrial fibrillation) or M6547 (Radiofrequency Ablation of Atrial fibrillation Through Intracardiac Electrophysiologic 3-Dimensional Mapping) |
| Cardioversion | Defined from diagnosis of AF plus claims for cardioversion | ICD-10: I48  Claim codes:M5880 |

*For greater accuracy, either one diagnosis during hospitalisation or more than twice at outpatient clinics was requited for the diagnosis.

eGFR, estimated glomerular filtration rate; ICD-10, International Classification of Diseases-10th Revision.

**Supplemental Table S2.** List of 109 variables contributing to calculating the Hospital Frailty Risk Score.

| **ICD-10 Description** | **ICD-10**  **code** | **Points** |
| --- | --- | --- |
| Dementia in Alzheimer's disease | F00 | 7.1 |
| Hemiplegia | G81 | 4.4 |
| Alzheimer's disease | G30 | 4 |
| Sequelae of cerebrovascular disease (secondary codes) | I69 | 3.7 |
| Other symptoms and signs involving the nervous and musculoskeletal systems (R29.6 Tendency to fall) | R29 | 3.6 |
| Other disorders of urinary system (includes urinary tract infection and urinary incontinence) | N39 | 3.2 |
| Superficial injury of head | S00 | 3.2 |
| Delirium, not induced by alcohol and other psychoactive substances | F05 | 3.2 |
| Unspecified fall | W19 | 3.2 |
| Unspecified hematuria | R31 | 3 |
| Other bacterial agents as the cause of diseases classified to other chapters (secondary code) | B96 | 2.9 |
| Other symptoms and signs involving cognitive functions and awareness | R41 | 2.7 |
| Other cerebrovascular diseases | I67 | 2.6 |
| Convulsions, not elsewhere classified | R56 | 2.6 |
| Abnormalities of gait and mobility | R26 | 2.6 |
| Somnolence, stupor and coma | R40 | 2.5 |
| Intracranial injury | S06 | 2.4 |
| Complications of genitourinary prosthetic devices, implants and grafts | T83 | 2.4 |
| Other disorders of fluid, electrolyte and acid base balance | E87 | 2.3 |
| Other joint disorders, not elsewhere classified | M25 | 2.3 |
| Volume depletion | E86 | 2.3 |
| Fracture of shoulder and upper arm | S42 | 2.3 |
| Senility | R54 | 2.2 |
| Unspecified dementia | F03 | 2.1 |
| Care involving use of rehabilitation procedures | Z50 | 2.1 |
| Other fall on same level | W18 | 2.1 |
| Cellulitis | L03 | 2 |
| Vascular dementia | F01 | 2 |
| Superficial injury of lower leg | S80 | 2 |
| Problems related to medical facilities and other health care | Z75 | 2 |
| Deficiency of other B group vitamins | E53 | 1.9 |
| Blindness and low vision | H54 | 1.9 |
| Other functional intestinal disorders | K59 | 1.8 |
| Fracture of rib(s), sternum and thoracic spine | S22 | 1.8 |
| Syncope and collapse | R55 | 1.8 |
| Acute renal failure | N17 | 1.8 |
| Parkinson's disease | G20 | 1.8 |
| Problems related to social environment | Z60 | 1.8 |
| Decubitus ulcer | L89 | 1.7 |
| Carrier of infectious disease | Z22 | 1.7 |
| Streptococcus and staphylococcus as the cause of diseases classified to other chapters | B95 | 1.7 |
| Other septicemia | A41 | 1.6 |
| Duodenal ulcer | K26 | 1.6 |
| Hypotension | I95 | 1.6 |
| Unspecified renal failure | N19 | 1.6 |
| Ulcer of lower limb, not elsewhere classified | L97 | 1.6 |
| Other symptoms and signs involving general sensations and perceptions | R44 | 1.6 |
| Epilepsy | G40 | 1.5 |
| Other arthrosis | M19 | 1.5 |
| Respiratory failure, not elsewhere classified | J96 | 1.5 |
| Personal history of other diseases and conditions | Z87 | 1.5 |
| Exposure to unspecified factor | X59 | 1.5 |
| Osteoporosis without pathological fracture | M81 | 1.4 |
| Abnormal results of function studies | R94 | 1.4 |
| Fracture of lumbar spine and pelvis | S32 | 1.4 |
| Chronic renal failure | N18 | 1.4 |
| Fracture of femur | S72 | 1.4 |
| Other disorders of pancreatic internal secretion | E16 | 1.4 |
| Other disorders of kidney and ureter, not elsewhere classified | N28 | 1.3 |
| Retention of urine | R33 | 1.3 |
| Unknown and unspecified causes of morbidity | R69 | 1.3 |
| Transient cerebral ischemic attacks and related syndromes | G45 | 1.2 |
| Other degenerative diseases of nervous system, not elsewhere classified | G31 | 1.2 |
| Unspecified urinary incontinence | R32 | 1.2 |
| Symptoms and signs involving emotional state | R45 | 1.2 |
| Other and unspecified injuries of head | S09 | 1.2 |
| Nosocomial condition | Y95 | 1.2 |
| Pneumonia, organism unspecified | J18 | 1.1 |
| Diarrhea and gastroenteritis of presumed infectious origin | A09 | 1.1 |
| Other soft tissue disorders, not elsewhere classified | M79 | 1.1 |
| Open wound of head | S01 | 1.1 |
| Other bacterial intestinal infections | A04 | 1.1 |
| Fall involving bed | W06 | 1.1 |
| Problems related to care-provider dependency | Z74 | 1.1 |
| Speech disturbances, not elsewhere classified | R47 | 1 |
| Pneumonitis due to solids and liquids | J69 | 1 |
| Artificial opening status | Z93 | 1 |
| Vitamin D deficiency | E55 | 1 |
| Gangrene, not elsewhere classified | R02 | 1 |
| Thyrotoxicosis [hyperthyroidism] | E05 | 0.9 |
| Symptoms and signs concerning food and fluid intake | R63 | 0.9 |
| Other hearing loss | H91 | 0.9 |
| Scoliosis | M41 | 0.9 |
| Fall on same level from slipping, tripping and stumbling | W01 | 0.9 |
| Fall on and from stairs and steps | W10 | 0.9 |
| Cerebral Infarction | I63 | 0.8 |
| Other diseases of digestive system | K92 | 0.8 |
| Dysphagia | R13 | 0.8 |
| Osteoporosis with pathological fracture | M80 | 0.8 |
| Agent resistant to penicillin and related antibiotics | U80 | 0.8 |
| Dependence on enabling machines and devices | Z99 | 0.8 |
| Abnormalities of heart beat | R00 | 0.7 |
| Calculus of kidney and ureter | N20 | 0.7 |
| Mental and behavioral disorders due to use of alcohol | F10 | 0.7 |
| Unspecified acute lower respiratory infection | J22 | 0.7 |
| Other medical procedures as the cause of abnormal reaction of the patient | Y84 | 0.7 |
| Other abnormal findings of blood chemistry | R79 | 0.6 |
| Problems related to life-management difficulty | Z73 | 0.6 |
| Spinal stenosis (secondary code only) | M48 | 0.5 |
| Depressive episode | F32 | 0.5 |
| Open wound of forearm | S51 | 0.5 |
| Personal history of risk-factors, not elsewhere classified | Z91 | 0.5 |
| Other anemia | D64 | 0.4 |
| Disorders of mineral metabolism | E83 | 0.4 |
| Polyarthrosis | M15 | 0.4 |
| Other local infections of skin and subcutaneous tissue | L08 | 0.4 |
| Nausea and vomiting | R11 | 0.3 |
| Other noninfective gastroenteritis and colitis | K52 | 0.3 |
| Fever of unknown origin | R50 | 0.1 |

ICD-10, International Classification of Diseases-10th Revision.

**Supplemental Table S3.** Definitions and codes used for study outcomes.

| **Outcomes** | **Definitions** | **Codes or conditions** | **PPV** |
| --- | --- | --- | --- |
| **Primary composite outcomes** | |  |  |
| Ischaemic stroke | Defined from admission diagnosis with concomitant imaging studies of the brain or related death | ICD-10: I63, I64 | 90.6%*  (2347/2591) |
| Hospitalisation owing to heart failure | Defined from principal or first secondary admission diagnoses of heart failure | ICD-10: I11.0, I50, I97.1 | 82.1%*  (110/134) |
| Acute myocardial infarction | Defined from admission diagnosis of acute myocardial infarction concurrently with coronary angiography or related death | ICD-10: I21, I22 | 86.5%†  (4054/4688) |
| **Safety outcomes** | |  |  |
| Intracranial bleeding | Defined from admission diagnosis with concomitant imaging studies of the brain or related death | ICD-10: I60-I62 | 87.5%*  (286/327) |
| Gastrointestinal bleeding | Defined from admission diagnosis or related death | ICD-10: K25-28 (subcodes 0-2 and 4-6 only), K62.5, K92.0, K92.1, K92.2, I85.0, I98.3 | 92.0%‡  (184/200) |
| Serious adverse events related to rhythm control | |  |  |
| Cardiac tamponade | Defined from claims for pericardiocentesis | Claim codes:C8060, C8061 | - |
| Syncope | Defined from either one diagnosis during hospitalisation or more than twice at outpatient clinics | ICD-10: R55.x | - |
| Sick sinus syndrome | Defined from either one diagnosis during hospitalisation or more than twice at outpatient clinics | ICD-10: I495. | 91.1%*  (307/337) |
| Atrioventricular block | Defined from either one diagnosis during hospitalisation or more than twice at outpatient clinics | ICD-10: I44.1, I44.2, I44.3, I45.3, I45.8, I45.9 | 95.7%*  (264/276) |
| Pacemaker implantation | Defined from claims for pacemaker implantation | Claim codes: O2003, O2004, O0203, O0204, O0205, O0206, O0207 | - |
| Sudden cardiac arrest | Defined from admission diagnosis or related death§ | ICD-10: I46, I49.0 | 80.2%⁑  (586/731) |

PPV was represented as % (number of true positive cases / number of examined cases).
*Validated in a study by Kim, D. et al. (Treatment timing and the effects of rhythm control strategy in patients with atrial fibrillation: nationwide cohort study. *BMJ* 2021;373:n991).

†Validated in a study by Lee, HY. et al. (Atrial fibrillation and the risk of myocardial infarction: a nation-wide propensity-matched study. *Sci Rep* 2017;7(1):12716).

§To avoid erroneous inclusion of the patients with non-cardiac arrest, we excluded the patient with sudden arrest diagnosis accompanied by respiratory arrest (R09.0, R09.2), gastrointestinal bleeding (I85.0, K25.0, K25.4, K26.0, K26.4, K27.0, K27.4, K92.0-K92.2), brain haemorrhage (I60.x-I62.x, S06.4-S06.6), septic shock (A41.9, R57.2), pregnancy and delivery (O00-O99), diabetic ketoacidosis (E14.1), anaphylaxis (T78.2), and accidents including suicide (T71, T75.1, T36-T65, V80-V89, W76.x, X60-X84).

⁑Validated in a study by Kim, IJ. et al. (Relationship Between Anemia and the Risk of Sudden Cardiac Arrest - A Nationwide Cohort Study in South Korea. Circ J 2018;82(12):2962-9).

ICD-10, International Classification of Diseases-10th Revision; PPV, positive predictive value.

**Supplemental Table S4.** Baseline characteristics after overlap weighting.

|  | **Non-frail** | | | **Intermediate-frail** | | | **High-frail** | | |
| --- | --- | --- | --- | --- | --- | --- | --- | --- | --- |
| **Variables** | Rhythm  control  (n=2165.2) | Rate  control  (n=2165.2) | ASD | Rhythm  control  (n =848.2) | Rate  control  (n=848.2) | ASD | Rhythm  control  (n=177.2) | Rate  control  (n=177.2) | ASD |
| **Sociodemographic** | | | | | | | | | |
| Age, years | 72 (69-77) | 72 (68-77) | <0.1 | 74 (70-79) | 75 (71-80) | <0.1 | 77 (71-82) | 76 (72-82) | <0.1 |
| Male | 1109.3 (51.2) | 1109.3 (51.2) | <0.1 | 395.9 (46.7) | 395.9 (46.7) | <0.1 | 70.7 (39.9) | 70.7 (39.9) | <0.1 |
| AF duration, months | 0.0 (0.0-0.8) | 0.0 (0.0-0.3) | <0.1 | 0.9 (0.0-0.8) | 0.0 (0.0-0.5) | <0.1 | 0.0 (0.0-1.5) | 0.0 (0.0-1.3) | <0.1 |
| High tertile of income | 1025.6 (47.4) | 1025.6 (47.4) | <0.1 | 392.1 (46.2) | 392.1 (46.2) | <0.1 | 87.5 (49.4) | 87.5 (49.4) | <0.1 |
| Number of OPD visits ≥12/year | 1846.5 (85.3) | 1846.5 (85.3) | <0.1 | 696.3 (82.1) | 696.3 (82.1) | <0.1 | 125.2 (70.7) | 125.2 (70.7) | <0.1 |
| Living in metropolitan areas | 1000.6 (46.2) | 1000.6 (46.2) | <0.1 | 348.4 (41.1) | 348.4 (41.1) | <0.1 | 66.8 (37.7) | 66.8 (37.7) | <0.1 |
| Level of care initiating treatment | | | | | | | | | |
| Tertiary | 1096.9 (50.7) | 1096.9 (50.7) | <0.1 | 392.6 (46.3) | 392.6 (46.3) | <0.1 | 75.4 (42.6) | 75.4 (42.6) | <0.1 |
| Secondary | 914.4 (42.2) | 914.4 (42.2) | <0.1 | 433.9 (51.2) | 433.9 (51.2) | <0.1 | 98.6 (55.7) | 98.6 (55.7) | <0.1 |
| Primary | 153.8 (7.1) | 153.8 (7.1) | <0.1 | 21.7 (2.6) | 21.7 (2.6) | <0.1 | 3.1 (1.8) | 3.1 (1.8) | <0.1 |
| **Risk scores** | | | | | | | | | |
| CHA_2_DS_2_-VASc score | 4 (3-5) | 4 (3-5) | <0.1 | 5 (4-6) | 5 (4-6) | <0.1 | 6 (5-7) | 6 (5-7) | <0.1 |
| mHAS-BLED score* | 3 (2-3) | 3 (2-3) | <0.1 | 3 (2-4) | 3 (2-4) | <0.1 | 3 (3-4) | 3 (3-4) | <0.1 |
| Charlson comorbidity index | 3 (2-5) | 3 (1-5) | <0.1 | 5 (3-7) | 5 (3-7) | <0.1 | 7 (5-9) | 7 (5-9) | <0.1 |
| Hospital Frailty Risk score | 0.8 (0.0-2.4) | 0.8 (0.0-2.4) | <0.1 | 8.0 (6.3-10.4) | 8.0 (6.2-10.5) | <0.1 | 19.0  (16.7-23.0) | 18.9  (16.5-23.5) | <0.1 |
| **Medical history** | | | | | | | | | |
| Heart failure | 997.3 (46.1) | 997.3 (46.1) | <0.1 | 432.8 (51.0) | 432.8 (51.0) | <0.1 | 100.8 (56.9) | 100.8 (56.9) | <0.1 |
| Hx of admission owing to heart failure | 291.5 (13.5) | 291.5 (13.5) | <0.1 | 141.2 (16.6) | 141.2 (16.6) | <0.1 | 28.2 (15.9) | 28.2 (15.9) | <0.1 |
| Hypertension | 1664.3 (76.9) | 1664.3 (76.9) | <0.1 | 697.1 (82.2) | 697.1 (82.2) | <0.1 | 156.8 (88.5) | 156.8 (88.5) | <0.1 |
| Diabetes | 514.8 (23.8) | 514.8 (23.8) | <0.1 | 285.8 (33.7) | 285.8 (33.7) | <0.1 | 76.8 (43.3) | 76.8 (43.3) | <0.1 |
| Dyslipidaemia | 1559.1 (72.0) | 1559.1 (72.0) | <0.1 | 700.4 (82.6) | 700.4 (82.6) | <0.1 | 155.1 (87.5) | 155.1 (87.5) | <0.1 |
| Ischemic stroke | 497.2 (23.0) | 497.2 (23.0) | <0.1 | 455.4 (53.7) | 455.4 (53.7) | <0.1 | 135.7 (76.6) | 135.7 (76.6) | <0.1 |
| Transient ischaemic attack | 150.2 (6.9) | 150.2 (6.9) | <0.1 | 124.6 (14.7) | 124.6 (14.7) | <0.1 | 36.7 (20.7) | 36.7 (20.7) | <0.1 |
| Haemorrhagic stroke | 19.6 (0.9) | 19.6 (0.9) | <0.1 | 34.7 (4.1) | 34.7 (4.1) | <0.1 | 19.7 (11.1) | 19.7 (11.1) | <0.1 |
| Myocardial infarction | 155.4 (7.2) | 155.4 (7.2) | <0.1 | 103.7 (12.2) | 103.7 (12.2) | <0.1 | 30.4 (17.2) | 30.4 (17.2) | <0.1 |
| Peripheral arterial disease | 254.4 (11.7) | 254.4 (11.7) | <0.1 | 129.5 (15.3) | 129.5 (15.3) | <0.1 | 39.3 (22.2) | 39.3 (22.2) | <0.1 |
| Valvular heart disease | 195.4 (9.0) | 195.4 (9.0) | <0.1 | 61.7 (7.3) | 61.7 (7.3) | <0.1 | 8.1 (4.6) | 8.1 (4.6) | <0.1 |
| Chronic kidney disease | 75.4 (3.5) | 75.4 (3.5) | <0.1 | 61.6 (7.3) | 61.6 (7.3) | <0.1 | 25.4 (14.3) | 25.4 (14.3) | <0.1 |
| Proteinuria | 112.5 (5.2) | 112.5 (5.2) | <0.1 | 50.9 (6.0) | 50.9 (6.0) | <0.1 | 11.9 (6.7) | 11.9 (6.7) | <0.1 |
| Hyperthyroidism | 194.3 (9.0) | 194.3 (9.0) | <0.1 | 103.2 (12.2) | 103.2 (12.2) | <0.1 | 23.9 (13.5) | 23.9 (13.5) | <0.1 |
| Hypothyroidism | 204.1 (9.4) | 204.1 (9.4) | <0.1 | 109.6 (12.9) | 109.6 (12.9) | <0.1 | 28.4 (16.0) | 28.4 (16.0) | <0.1 |
| Malignancy | 414.1 (19.1) | 414.1 (19.1) | <0.1 | 246.7 (29.1) | 246.7 (29.1) | <0.1 | 60.4 (34.1) | 60.4 (34.1) | <0.1 |
| COPD | 645.8 (29.8) | 645.8 (29.8) | <0.1 | 347.0 (40.9) | 347.0 (40.9) | <0.1 | 83.9 (47.3) | 83.9 (47.3) | <0.1 |
| Chronic liver disease | 717.5 (33.1) | 717.5 (33.1) | <0.1 | 355.9 (42.0) | 355.9 (42.0) | <0.1 | 79.4 (44.8) | 79.4 (44.8) | <0.1 |
| Hypertrophic cardiomyopathy | 40.9 (1.9) | 40.9 (1.9) | <0.1 | 12.8 (1.5) | 12.8 (1.5) | <0.1 | 3.0 (1.7) | 3.0 (1.7) | <0.1 |
| Osteoporosis | 710.7 (32.8) | 710.7 (32.8) | <0.1 | 423.5 (49.9) | 423.5 (49.9) | <0.1 | 116.1 (65.5) | 116.1 (65.5) | <0.1 |
| Sleep apnea | 5.7 (0.3) | 5.7 (0.3) | <0.1 | 1.4 (0.2) | 1.4 (0.2) | <0.1 | 0.0 (0.0) | 0.0 (0.0) | <0.1 |
| **Concurrent medication**† | | | | | | | | | |
| Oral anticoagulant | 2165.2 (100.0) | 2165.2 (100.0) | <0.1 | 848.2 (100.0) | 848.2 (100.0) | <0.1 | 177.2 (100.0) | 177.2 (100.0) | <0.1 |
| Warfarin | 1931.1 (89.2) | 1931.1 (89.2) | <0.1 | 729.4 (86.0) | 729.4 (86.0) | <0.1 | 148.5 (83.8) | 148.5 (83.8) | <0.1 |
| NOAC | 308.4 (14.2) | 308.4 (14.2) | <0.1 | 163.0 (19.2) | 163.0 (19.2) | <0.1 | 37.1 (20.9) | 37.1 (20.9) | <0.1 |
| Beta-blocker | 1344.4 (62.1) | 1344.4 (62.1) | <0.1 | 528.6 (62.3) | 528.6 (62.3) | <0.1 | 107.0 (60.4) | 107.0 (60.4) | <0.1 |
| Non-DHP CCB | 403.9 (18.7) | 403.9 (18.7) | <0.1 | 149.7 (17.6) | 149.7 (17.6) | <0.1 | 35.0 (19.8) | 35.0 (19.8) | <0.1 |
| Digoxin | 456.1 (21.1) | 456.1 (21.1) | <0.1 | 157.1 (18.5) | 157.1 (18.5) | <0.1 | 36.2 (20.4) | 36.2 (20.4) | <0.1 |
| Aspirin | 592.5 (27.4) | 592.5 (27.4) | <0.1 | 205.2 (24.2) | 205.2 (24.2) | <0.1 | 38.0 (21.5) | 38.0 (21.5) | <0.1 |
| P2Y12 inhibitor | 198.2 (9.2) | 198.2 (9.2) | <0.1 | 103.4 (12.2) | 103.4 (12.2) | <0.1 | 28.2 (15.9) | 28.2 (15.9) | <0.1 |
| Statin | 817.2 (37.7) | 817.2 (37.7) | <0.1 | 391.4 (46.1) | 391.4 (46.1) | <0.1 | 87.3 (49.3) | 87.3 (49.3) | <0.1 |
| DHP CCB | 362.2 (16.7) | 362.2 (16.7) | <0.1 | 162.3 (19.1) | 162.3 (19.1) | <0.1 | 37.2 (21.0) | 37.2 (21.0) | <0.1 |
| ACEI/ARB | 1216.4 (56.2) | 1216.4 (56.2) | <0.1 | 439.9 (51.9) | 439.9 (51.9) | <0.1 | 91.2 (51.5) | 91.2 (51.5) | <0.1 |
| Loop/thiazide diuretics | 1105.9 (51.1) | 1105.9 (51.1) | <0.1 | 407.3 (48.0) | 407.3 (48.0) | <0.1 | 78.8 (44.5) | 78.8 (44.5) | <0.1 |
| K+ sparing diuretics | 405.4 (18.7) | 405.4 (18.7) | <0.1 | 144.7 (17.1) | 144.7 (17.1) | <0.1 | 29.7 (16.7) | 29.7 (16.7) | <0.1 |
| Alpha-blocker | 58.7 (2.7) | 58.7 (2.7) | <0.1 | 26.4 (3.1) | 26.4 (3.1) | <0.1 | 5.2 (3.0) | 5.2 (3.0) | <0.1 |

Values are presented as median (interquartile range) or n (%).

*Modified HAS-BLED = hypertension, 1 point: >65 years old, 1 point: stroke history, 1 point: bleeding history or predisposition, 1 point: liable international normalised ratio, not assessed: ethanol or drug abuse, 1 point: drug predisposing to bleeding, 1 point.

†Defined as a prescription fill of >90 days within the 180-day after the first prescription for rhythm- or rate-control drugs or the performance of an ablation procedure for AF.

AAD, antiarrhythmic drug; ACEI, angiotensin converting enzyme inhibitor; AF, atrial fibrillation; ARB, angiotensin II receptor blocker; ASD, absolute standardised difference; COPD, chronic obstructive pulmonary disease; DHP, dihydropyridine; NOAC, non-vitamin K antagonist oral anticoagulant; OPD, outpatient department

**Supplemental Table S5.** Baseline characteristics of overall patients undergoing rhythm- and rate-control treatments before and after propensity overlap weighting

|  | **Before overlap weighting** | | | | **After overlap weighting** | | |
| --- | --- | --- | --- | --- | --- | --- | --- |
| **Variables** | Rhythm  control  (n=9534) | Rate  control  (n=11077) | ASD (%) | | Rhythm control  (n=3221.8) | Rate  control  (n=3221.8) | ASD (%) |
| Age (years) | 73 (69-77) | 74 (69-79) | 20.7 | | 73 (69-78) | 73 (69-78) | <0.1 |
| 65-74 | 5878 (61.7) | 5908 (53.3) | 16.9 | | 1845.6 (57.3) | 1845.6 (57.3) | <0.1 |
| ≥75 | 3656 (38.3) | 5169 (46.7) | 16.9 | | 1376.2 (42.7) | 1376.2 (42.7) | <0.1 |
| Male | 4707 (49.4) | 5308 (47.9) | 2.9 | | 1594.5 (49.5) | 1594.5 (49.5) | <0.1 |
| AF duration (months) | 0.0 (0.0-1.2) | 0.0 (0.0-0.0) | 26.9 | | 0.0 (0.0-0.8) | 0.0 (0.0-0.4) | <0.1 |
| Enrolment year: |  |  |  | |  |  |  |
| 2005 | 425 (4.5) | 1242 (11.2) | 25.3 | 200.6 (6.2) | | 200.6 (6.2) | <0.1 |
| 2006 | 465 (4.9) | 1019 (9.2) | 17.0 | 202.7 (6.3) | | 202.7 (6.3) | <0.1 |
| 2007 | 456 (4.8) | 794 (7.2) | 10.1 | 189.5 (5.9) | | 189.5 (5.9) | <0.1 |
| 2008 | 458 (4.8) | 851 (7.7) | 11.9 | 203.8 (6.3) | | 203.8 (6.3) | <0.1 |
| 2009 | 522 (5.5) | 717 (6.5) | 4.2 | 190.5 (5.9) | | 190.5 (5.9) | <0.1 |
| 2010 | 649 (6.8) | 753 (6.8) | <0.1 | 222.0 (6.9) | | 222.0 (6.9) | <0.1 |
| 2011 | 823 (8.6) | 808 (7.3) | 4.9 | 254.4 (7.9) | | 254.4 (7.9) | <0.1 |
| 2012 | 952 (10.0) | 923 (8.3) | 5.7 | 309.3 (9.6) | | 309.3 (9.6) | <0.1 |
| 2013 | 1247 (13.1) | 1138 (10.3) | 8.7 | 397.0 (12.3) | | 397.0 (12.3) | <0.1 |
| 2014 | 1475 (15.5) | 1155 (10.4) | 15.1 | 424.1 (13.2) | | 424.1 (13.2) | <0.1 |
| 2015 | 2062 (21.6) | 1677 (15.1) | 16.8 | 627.9 (19.5) | | 627.9 (19.5) | <0.1 |
| High tertile of income | 4894 (51.3) | 4595 (41.5) | 19.8 | 1519.5 (47.2) | | 1519.5 (47.2) | <0.1 |
| Number of OPD visits ≥12/year | 8374 (87.8) | 8623 (77.8) | 26.7 | 2693.8 (83.6) | | 2693.8 (83.6) | <0.1 |
| Living in metropolitan areas | 4530 (47.5) | 4474 (40.4) | 14.4 | 1429.3 (44.4) | | 1429.3 (44.4) | <0.1 |
| Level of care initiating treatment |  |  |  |  | |  |  |
| Tertiary | 5624 (59.0) | 4326 (39.1) | 40.7 | 1582.9 (49.1) | | 1582.9 (49.1) | <0.1 |
| Secondary | 3554 (37.3) | 5726 (51.7) | 29.3 | 1458.2 (45.3) | | 1458.2 (45.3) | <0.1 |
| Primary | 356 (3.7) | 1025 (9.3) | 22.5 | 180.7 (5.6) | | 180.7 (5.6) | <0.1 |
| CHA_2_DS_2_-VASc score | 4 (3-6) | 4 (3-5) | 18.7 | 4 (3-6) | | 4 (3-6) | <0.1 |
| mHAS-BLED score* | 3 (2-4) | 2 (2-3) | 38.0 | 3 (2-3) | | 3 (2-3) | <0.1 |
| Charlson comorbidity Index | 4 (2-6) | 3 (1-4) | 49.9 | 4 (2-5) | | 3 (2-5) | <0.1 |
| Hospital frailty risk score | 2.3 (0.0-6.2) | 1.8 (0.0-5.6) | 7.8 | 2.3 (0.0-6.3) | | 2.3 (0.0-6.3) | <0.1 |
| Medical history |  |  |  |  | |  |  |
| Heart failure | 4407 (46.2) | 5581 (50.4) | 8.3 | 1549.3 (48.1) | | 1549.3 (48.1) | <0.1 |
| Previous hospitalisation for heart failure | 1238 (13.0) | 1750 (15.8) | 8.0 | 469.6 (14.6) | | 469.6 (14.6) | <0.1 |
| Hypertension | 8095 (84.9) | 7176 (64.8) | 47.7 | 2542.9 (78.9) | | 2542.9 (78.9) | <0.1 |
| Diabetes | 2875 (30.2) | 2496 (22.5) | 17.4 | 887.8 (27.6) | | 887.8 (27.6) | <0.1 |
| Dyslipidaemia | 7882 (82.7) | 6887 (62.2) | 47.1 | 2437.6 (75.7) | | 2437.6 (75.7) | <0.1 |
| Ischaemic stroke | 3120 (32.7) | 3576 (32.3) | 0.9 | 1099.4 (34.1) | | 1099.4 (34.1) | <0.1 |
| Transient ischaemic attack | 1113 (11.7) | 778 (7.0) | 16.0 | 313.4 (9.7) | | 313.4 (9.7) | <0.1 |
| Intracranial bleeding | 200 (2.1) | 236 (2.1) | 0.2 | 74.4 (2.3) | | 74.4 (2.3) | <0.1 |
| Myocardial infarction | 1043 (10.9) | 780 (7.0) | 13.7 | 293.2 (9.1) | | 293.2 (9.1) | <0.1 |
| Peripheral arterial disease | 1577 (16.5) | 1013 (9.1) | 22.2 | 428.8 (13.3) | | 428.8 (13.3) | <0.1 |
| Valvular heart disease | 776 (8.1) | 1064 (9.6) | 5.2 | 269.5 (8.4) | | 269.5 (8.4) | <0.1 |
| Chronic kidney disease | 631 (6.6) | 379 (3.4) | 14.7 | 165.6 (5.1) | | 165.6 (5.1) | <0.1 |
| Proteinuria | 570 (6.0) | 510 (4.6) | 6.1 | 177.1 (5.5) | | 177.1 (5.5) | <0.1 |
| Hyperthyroidism | 1231 (12.9) | 866 (7.8) | 16.8 | 327.0 (10.2) | | 327.0 (10.2) | <0.1 |
| Hypothyroidism | 1313 (13.8) | 847 (7.6) | 19.9 | 344.8 (10.7) | | 344.8 (10.7) | <0.1 |
| Malignancy | 2392 (25.1) | 2070 (18.7) | 15.5 | 730.3 (22.7) | | 730.3 (22.7) | <0.1 |
| COPD | 3358 (35.2) | 3368 (30.4) | 10.3 | 1088.1 (33.8) | | 1088.1 (33.8) | <0.1 |
| Chronic liver disease | 3918 (41.1) | 3226 (29.1) | 25.3 | 1164.7 (36.2) | | 1164.7 (36.2) | <0.1 |
| Hypertrophic cardiomyopathy | 252 (2.6) | 127 (1.1) | 11.0 | 57.8 (1.8) | | 57.8 (1.8) | <0.1 |
| Osteoporosis | 4012 (42.1) | 3789 (34.2) | 16.3 | 1261.0 (39.1) | | 1261.0 (39.1) | <0.1 |
| Sleep apnea | 32 (0.3) | 16 (0.1) | 3.9 | 7.7 (0.2) | | 7.7 (0.2) | <0.1 |
| Concurrent drugs† |  |  |  |  | |  |  |
| Oral anticoagulant | 9534 (100.0) | 11077 (100.0) | <0.1 | 3221.8 (100.0) | | 3221.8 (100.0) | <0.1 |
| Warfarin | 8138 (85.4) | 10097 (91.2) | 18.1 | 2836.4 (88.0) | | 2836.4 (88.0) | <0.1 |
| NOAC | 1800 (18.9) | 1296 (11.7) | 20.1 | 512.1 (15.9) | | 512.1 (15.9) | <0.1 |
| Beta blocker | 4202 (44.1) | 6922 (62.5) | 37.6 | 1999.7 (62.1) | | 1999.7 (62.1) | <0.1 |
| Non-DHP CCB | 1325 (13.9) | 1946 (17.6) | 10.1 | 594.9 (18.5) | | 594.9 (18.5) | <0.1 |
| Digoxin | 878 (9.2) | 4862 (43.9) | 85.4 | 655.6 (20.3) | | 655.6 (20.3) | <0.1 |
| Aspirin | 2591 (27.2) | 2656 (24.0) | 7.3 | 844.1 (26.2) | | 844.1 (26.2) | <0.1 |
| P2Y12 inhibitor | 1019 (10.7) | 918 (8.3) | 8.2 | 333.8 (10.4) | | 333.8 (10.4) | <0.1 |
| Statin | 4070 (42.7) | 3845 (34.7) | 16.4 | 1309.3 (40.6) | | 1309.3 (40.6) | <0.1 |
| DHP-CCB | 2229 (23.4) | 1455 (13.1) | 26.8 | 566.9 (17.6) | | 566.9 (17.6) | <0.1 |
| ACEI/ARB | 5333 (55.9) | 6203 (56.0) | 0.1 | 1767.2 (54.9) | | 1767.2 (54.9) | <0.1 |
| Loop/thiazide diuretics | 4170 (43.7) | 6482 (58.5) | 29.9 | 1609.3 (49.9) | | 1609.3 (49.9) | <0.1 |
| K+ sparing diuretics | 1347 (14.1) | 2775 (25.1) | 27.8 | 587.0 (18.2) | | 587.0 (18.2) | <0.1 |
| Alpha blocker | 277 (2.9) | 328 (3.0) | 0.3 | 91.8 (2.8) | | 91.8 (2.8) | <0.1 |

Values are presented as median (interquartile range) or n (%).

*Modified HAS-BLED = hypertension, 1 point: >65 years old, 1 point: stroke history, 1 point: bleeding history or predisposition, 1 point: liable international normalised ratio, not assessed: ethanol or drug abuse, 1 point: drug predisposing to bleeding, 1 point.

†Defined as a prescription fill of >90 days within 180 days after the first prescription for rhythm- or rate-control drugs or the performance of an ablation procedure for AF.

AAD, antiarrhythmic drug; ACEI, angiotensin converting enzyme inhibitor; AF, atrial fibrillation; ARB, angiotensin II receptor blocker; ASD, absolute standardised difference; COPD, chronic obstructive pulmonary disease; DHP, dihydropyridine; NOAC, non-vitamin K antagonist oral anticoagulant; OPD, outpatient department.

**Supplemental Table S6.** Baseline characteristics after propensity score matching.

|  | **Non-frail** | | | **Intermediate-frail** | | | **High-frail** | | |
| --- | --- | --- | --- | --- | --- | --- | --- | --- | --- |
| **Variables** | Rhythm  Control  (N=3063) | Rate  Control  (N=3063) | ASD | Rhythm  Control  (N=1224) | Rate  Control  (N=1224) | ASD | Rhythm  Control  (N=242) | Rate  Control  (N=242) | ASD |
| **Sociodemographic** | | | | | | | | | |
| Age, years | 73 (69-77) | 72 (69-77) | 0.9% | 75 (71-79) | 75 (70-79) | 0.7% | 77 (72-82) | 77 (72-82) | 6.7% |
| Male | 1581 (51.6) | 1565 (51.1) | 1.0% | 565 (46.2) | 577 (47.1) | 2.0% | 101 (41.7) | 99 (40.9) | 1.7% |
| AF duration, months | 0.0 (0.0-0.7) | 0.0 (0.0-0.3) | 2.2% | 0.0 (0.0-0.7) | 0.0 (0.0-0.5) | 0.6% | 0.0 (0.0-1.4) | 0.0 (0.0-0.9) | 4.0% |
| High tertile of income | 1450 (47.3) | 1457 (47.6) | 0.5% | 565 (46.2) | 568 (46.4) | 0.5% | 125 (51.7) | 120 (49.6) | 4.1% |
| Number of OPD visits ≥12/year | 2610 (85.2) | 2599 (84.9) | 1.0% | 1001 (81.8) | 996 (81.4) | 1.1% | 168 (69.4) | 168 (69.4) | <0.1% |
| Living in metropolitan areas | 1398 (45.6) | 1417 (46.3) | 1.2% | 506 (41.3) | 497 (40.6) | 1.5% | 90 (37.2) | 98 (40.5) | 6.8% |
| Level of care initiating treatment | | | | | | | | | |
| Tertiary | 1544 (50.4) | 1554 (50.7) | 0.7% | 572 (46.7) | 568 (46.4) | 0.7% | 99 (40.9) | 105 (43.4) | 5.0% |
| Secondary | 1316 (43.0) | 1304 (42.6) | 0.8% | 621 (50.7) | 628 (51.3) | 1.1% | 139 (57.4) | 130 (53.7) | 7.5% |
| Primary | 203 (6.6) | 205 (6.7) | 0.3% | 31 (2.5) | 28 (2.3) | 1.6% | 4 (1.7) | 7 (2.9) | 8.3% |
| **Risk scores** | | | | | | | | | |
| CHA_2_DS_2_-VASc score | 4 (3-5) | 4 (3-5) | 0.4% | 5 (4-6) | 5 (4-6) | 0.2% | 6 (5-7) | 6 (5-7) | 0.8% |
| mHAS-BLED score* | 3 (2-3) | 3 (2-3) | 3.0% | 3 (2-4) | 3 (2-4) | 1.7% | 3 (3-4) | 3 (3-4) | 2.1% |
| Charlson comorbidity index | 3 (2-4) | 3 (1-5) | 0.6% | 5 (3-6) | 4 (3-7) | 0.4% | 6 (5-8) | 6 (4-9) | 0.1% |
| Hospital Frailty Risk score | 0.80 (0.0-2.3) | 0.8 (0.0-2.4) | 0.3% | 8.0 (6.0-10.0) | 7.9 (6.2-10.4) | 3.0% | 18.9 (16.8-22.5) | 18.9 (16.5-22.6) | 3.1% |
| **Medical history** | | | | | | | | | |
| Heart failure | 1411 (46.1) | 1412 (46.1) | 0.1% | 618 (50.5) | 616 (50.3) | 0.3% | 140 (57.9) | 131 (54.1) | 7.5% |
| Hx of admission owing to heart failure | 410 (13.4) | 409 (13.4) | 0.1% | 195 (15.9) | 185 (15.1) | 2.3% | 34 (14.0) | 38 (15.7) | 4.6% |
| Hypertension | 2362 (77.1) | 2383 (77.8) | 1.6% | 1001 (81.8) | 1005 (82.1) | 0.8% | 210 (86.8) | 211 (87.2) | 1.2% |
| Diabetes | 726 (23.7) | 740 (24.2) | 1.1% | 393 (32.1) | 392 (32.0) | 0.2% | 108 (44.6) | 105 (43.4) | 2.5% |
| Dyslipidaemia | 2200 (71.8) | 2219 (72.4) | 1.4% | 1013 (82.8) | 1000 (81.7) | 2.8% | 212 (87.6) | 211 (87.2) | 1.2% |
| Ischemic stroke | 699 (22.8) | 695 (22.7) | 0.3% | 655 (53.5) | 667 (54.5) | 2.0% | 182 (75.2) | 184 (76.0) | 1.9% |
| Transient ischaemic attack | 203 (6.6) | 203 (6.6) | <0.1% | 175 (14.3) | 173 (14.1) | 0.5% | 48 (19.8) | 44 (18.2) | 4.2% |
| Haemorrhagic stroke | 25 (0.8) | 27 (0.9) | 0.7% | 54 (4.4) | 47 (3.8) | 2.9% | 28 (11.6) | 29 (12.0) | 1.3% |
| Myocardial infarction | 223 (7.3) | 238 (7.8) | 1.9% | 141 (11.5) | 134 (10.9) | 1.8% | 36 (14.9) | 39 (16.1) | 3.4% |
| Peripheral arterial disease | 367 (12.0) | 356 (11.6) | 1.1% | 192 (15.7) | 182 (14.9) | 2.3% | 50 (20.7) | 52 (21.5) | 2.0% |
| Valvular heart disease | 265 (8.7) | 265 (8.7) | <0.1% | 79 (6.5) | 91 (7.4) | 3.9% | 8 (3.3) | 8 (3.3) | <0.1% |
| Chronic kidney disease | 115 (3.8) | 107 (3.5) | 1.4% | 94 (7.7) | 81 (6.6) | 4.1% | 34 (14.0) | 30 (12.4) | 4.9% |
| Proteinuria | 161 (5.3) | 150 (4.9) | 1.6% | 69 (5.6) | 74 (6.0) | 1.7% | 18 (7.4) | 17 (7.0) | 1.6% |
| Hyperthyroidism | 262 (8.6) | 268 (8.7) | 0.7% | 149 (12.2) | 155 (12.7) | 1.5% | 28 (11.6) | 26 (10.7) | 2.6% |
| Hypothyroidism | 284 (9.3) | 284 (9.3) | <0.1% | 162 (13.2) | 153 (12.5) | 2.2% | 37 (15.3) | 37 (15.3) | <0.1% |
| Malignancy | 585 (19.1) | 575 (18.8) | 0.8% | 343 (28.0) | 355 (29.0) | 2.2% | 83 (34.3) | 89 (36.8) | 5.2% |
| COPD | 897 (29.3) | 928 (30.3) | 2.2% | 508 (41.5) | 500 (40.8) | 1.3% | 116 (47.9) | 119 (49.2) | 2.5% |
| Chronic liver disease | 990 (32.3) | 1011 (33.0) | 1.5% | 512 (41.8) | 515 (42.1) | 0.5% | 107 (44.2) | 109 (45.0) | 1.7% |
| Hypertrophic cardiomyopathy | 57 (1.9) | 57 (1.9) | <0.1% | 18 (1.5) | 18 (1.5) | <0.1% | 5 (2.1) | 4 (1.7) | 3.1% |
| Osteoporosis | 1002 (32.7) | 993 (32.4) | 0.6% | 622 (50.8) | 602 (49.2) | 3.3% | 160 (66.1) | 156 (64.5) | 3.5% |
| Sleep apnea | 8 (0.3) | 8 (0.3) | <0.1% | 2 (0.2) | 2 (0.2) | <0.1% | 242 (100.0) | 242 (100.0) | <0.1% |
| **Concurrent medication**† | | | | | | | | | |
| Oral anticoagulant | 3063 (100.0) | 3063 (100.0) | <0.1% | 1224 (100.0) | 1224 (100.0) | <0.1% | 242 (100.0) | 242 (100.0) | <0.1% |
| Warfarin | 2732 (89.2) | 2735 (89.3) | 0.3% | 1060 (86.6) | 1067 (87.2) | 1.7% | 202 (83.5) | 203 (83.9) | 1.1% |
| NOAC | 436 (14.2) | 437 (14.3) | 0.1% | 227 (18.5) | 221 (18.1) | 1.3% | 54 (22.3) | 50 (20.7) | 5.0% |
| Beta-blocker | 2017 (65.9) | 1949 (63.6) | 4.6% | 803 (65.6) | 756 (61.8) | 8.0% | 149 (61.6) | 148 (61.2) | 0.8% |
| Non-DHP CCB | 585 (19.1) | 573 (18.7) | 1.0% | 214 (17.5) | 222 (18.1) | 1.7% | 49 (20.2) | 48 (19.8) | 1.0% |
| Digoxin | 580 (18.9) | 647 (21.1) | 5.5% | 209 (17.1) | 231 (18.9) | 4.7% | 48 (19.8) | 45 (18.6) | 3.1% |
| Aspirin | 835 (27.3) | 864 (28.2) | 2.1% | 279 (22.8) | 297 (24.3) | 3.5% | 51 (21.1) | 50 (20.7) | 1.0% |
| P2Y12 inhibitor | 281 (9.2) | 289 (9.4) | 0.9% | 143 (11.7) | 148 (12.1) | 1.3% | 37 (15.3) | 39 (16.1) | 2.3% |
| Statin | 1167 (38.1) | 1174 (38.3) | 0.5% | 567 (46.3) | 563 (46.0) | 0.7% | 116 (47.9) | 123 (50.8) | 5.8% |
| DHP CCB | 486 (15.9) | 501 (16.4) | 1.3% | 232 (19.0) | 236 (19.3) | 0.8% | 51 (21.1) | 50 (20.7) | 1.0% |
| ACEI/ARB | 1691 (55.2) | 1756 (57.3) | 4.3% | 637 (52.0) | 629 (51.4) | 1.3% | 120 (49.6) | 122 (50.4) | 1.7% |
| Loop/thiazide diuretics | 1566 (51.1) | 1593 (52.0) | 1.8% | 584 (47.7) | 570 (46.6) | 2.3% | 106 (43.8) | 109 (45.0) | 2.5% |
| K+ sparing diuretics | 574 (18.7) | 601 (19.6) | 2.2% | 206 (16.8) | 212 (17.3) | 1.3% | 37 (15.3) | 37 (15.3) | <0.1% |
| Alpha-blocker | 79 (2.6) | 85 (2.8) | 1.2% | 37 (3.0) | 39 (3.2) | 0.9% | 5 (2.1) | 7 (2.9) | 5.3% |

Values are presented as median (interquartile range) or n (%).

*Modified HAS-BLED = hypertension, 1 point: >65 years old, 1 point: stroke history, 1 point: bleeding history or predisposition, 1 point: liable international normalised ratio, not assessed: ethanol or drug abuse, 1 point: drug predisposing to bleeding, 1 point.

†Defined as a prescription fill of >90 days within the 180-day after the first prescription for rhythm- or rate-control drugs or the performance of an ablation procedure for AF.

AAD, antiarrhythmic drug; ACEI, angiotensin converting enzyme inhibitor; AF, atrial fibrillation; ARB, angiotensin II receptor blocker; ASD, absolute standardised difference; COPD, chronic obstructive pulmonary disease; DHP, dihydropyridine; NOAC, non-vitamin K antagonist oral anticoagulant; OPD, outpatient department.

**Supplemental Table S7.** Definitions of 30 falsification endpoints.

| **Falsification endpoints** | **Definitions** | **ICD-10 codes / other conditions** |
| --- | --- | --- |
| Influenza | Defined from diagnosis plus treatment | J09, J10, J11 / Treatment: Oseltamivir |
| Major fracture | Defined from diagnosis on inpatient or emergency department record | S72, S72.0, S72.1, S72.2, S12.0, S12.1, S12.2, S12.7, S12.9, S22.0, S22.1, S32.0, S32 |
| Urinary tract infection | Defined from diagnosis* | N30, N300, N309, N341, N342, N390 |
| Tuberculosis | Defined from diagnosis* | A15, A16, A17, A18, A19 |
| Syphilis | Defined from diagnosis* | A50, A51, A52, A53 |
| Viral enteritis | Defined from diagnosis* | A08 |
| Warts | Defined from diagnosis* | B07 |
| Stomach cancer | Defined from diagnosis* | C16 |
| Bone malignancy | Defined from diagnosis* | C40, C41, C90, C795 |
| Lymphoma | Defined from diagnosis* | C81, C82, C83, C84, C85 |
| Lipoma | Defined from diagnosis* | D17 |
| Carpal tunnel syndrome | Defined from diagnosis* | G560 |
| Hordeolum / chalazion | Defined from diagnosis* | H00 |
| Pterygium | Defined from diagnosis* | H110 |
| Otitis media | Defined from diagnosis* | H65, H66, H67 |
| Meniere's disease | Defined from diagnosis* | H810 |
| Acute appendicitis | Defined from diagnosis on inpatient or emergency department record | K35 |
| Diverticulitis of intestine | Defined from diagnosis* | K57 |
| Cholecystitis | Defined from diagnosis* | K81 |
| Urticaria | Defined from diagnosis* | L50 |
| Ingrowing nail | Defined from diagnosis* | L600 |
| Seropositive rheumatoid arthritis | Defined from diagnosis* | M05 |
| Gout | Defined from diagnosis* | M10 |
| Frozen shoulder | Defined from diagnosis* | M750 |
| Osteomyelitis | Defined from diagnosis* | M86 |
| Nausea and vomiting | Defined from diagnosis* | R11 |
| Dysuria | Defined from diagnosis* | R30 |
| Voice disturbances | Defined from diagnosis* | R49 |
| Seizure | Defined from diagnosis* | R56 |
| Anaphylaxis/Allergic reaction | Defined from diagnosis* | T78 |

*To ensure accuracy, diagnosis was established based on one inpatient or two outpatient records of ICD-10 codes in the database.

ICD-10, International Classification of Diseases-10th Revision.

**Supplemental Table S8.** Crossovers during the follow-up.

|  | **Overall** | | **Non-frail**  **(HFRS <5)** | | **Moderately-frail**  **(HFRS 5-15)** | | **Highly-frail (HFRS >15)** | |
| --- | --- | --- | --- | --- | --- | --- | --- | --- |
|  | **Rhythm control**  **(N=9534)** | **Rate**  **control**  **(N=11077)** | **Rhythm control**  **(N=6520)** | **Rhythm control**  **(N=7909)** | **Rhythm control**  **(N=2500)** | **Rhythm control**  **(N=2635)** | **Rhythm control**  **(N=514)** | **Rhythm control**  **(N=533)** |
| **Crossover** | To rate control | To rhythm  Control | To rate control | To rate control | To rate control | To rate control | To rate control | To rate control |
| **N (%)** | 5013 (52.6) | 1003 (9.1) | 3363 (51.6) | 791 (10.0) | 1351 (54.0) | 185 (7.0) | 299 (58.2) | 27 (5.1) |
| **Median days to crossover (interquartile range)** | 259  (20-819) | 572  (195-1257) | 296  (24-940) | 624  (229-1355) | 220  (11-685) | 421  (124-950) | 181  (15-508) | 329  (101-825) |

HFRS, Hospital Frailty Risk Score.

**Supplemental Table S9.** Outcomes in weighted patients undergoing rhythm or rate control in on-treatment analyses in which patients were censored patients were censored at the time of crossover between treatment modalities or discontinuation of treatment.

| **Outcome** | Number of events | Person-years | Event rate |  | | Number of events | | Person-years | Event rate | Absolute rate difference per 100 person-years  (95% CI) | | Weighted HR  (95% CI) | p value |
| --- | --- | --- | --- | --- | --- | --- | --- | --- | --- | --- | --- | --- | --- |
| ***Highly-frail (HFRS >15)*** | Rhythm control | | |  | | Rate control | | | |  | |  |  |
| Primary composite outcome | 40 | 200 | 19.8 |  | | 49 | | 234 | 21.1 | -1.3 (-9.8 to 7.2) | | 0.93 (0.70-1.25) | 0.648 |
| Components of primary outcome |  |  |  |  | |  | |  |  |  | |  |  |
| Cardiovascular death | 10 | 229 | 4.2 |  | | 16 | | 284 | 5.5 | -1.4 (-5.2 to 2.4) | | 0.74 (0.42-1.31) | 0.304 |
| Ischaemic stroke | 19 | 213 | 8.8 |  | | 25 | | 256 | 9.8 | -1.0 (-6.5 to 4.6) | | 0.87 (0.57-1.33) | 0.525 |
| Hospitalisation for heart failure | 16 | 215 | 7.6 |  | | 20 | | 263 | 7.5 | 0.1 (-4.9 to 5.0) | | 1.01 (0.63-1.61) | 0.973 |
| Acute myocardial infarction | 1 | 229 | 0.3 |  | | 4 | | 280 | 1.3 | -1.0 (-2.5 to 0.5) | | 0.23 (0.03-1.59) | 0.135 |
| ***Moderately-frail (HFRS 5-15)*** | Rhythm control | | | |  | | Rate control | | | |  | | |
| Primary composite outcome | 159 | 1398 | 11.4 |  | | 218 | | 1673 | 13.0 | -1.6 (-4.1 to 0.9) | | 0.83 (0.72-0.96) | 0.013 |
| Components of primary outcome |  |  |  |  | |  | |  |  |  | |  |  |
| Cardiovascular death | 31 | 1587 | 2.0 |  | | 37 | | 1996 | 1.8 | 0.1 (-0.8 to 1.0) | | 1.03 (0.73-1.45) | 0.760 |
| Ischaemic stroke | 70 | 1495 | 4.7 |  | | 107 | | 1834 | 5.9 | -1.2 (-2.7 to 0.4) | | 0.76 (0.62-0.95) | 0.014 |
| Hospitalisation for heart failure | 81 | 1476 | 5.5 |  | | 113 | | 1811 | 6.3 | -0.7 (-2.4 to 0.9) | | 0.83 (0.68-1.01) | 0.066 |
| Acute myocardial infarction | 7 | 1580 | 0.4 |  | | 11 | | 1987 | 0.5 | -0.1 (-0.6 to 0.4) | | 0.80 (0.42-1.54) | 0.503 |
| ***Non-frail (HFRS <5)*** | Rhythm control | | | |  | | Rate control | | | |  | | |
| Primary composite outcome | 328 | 4915 | 6.7 |  | | 533 | | 6224 | 8.6 | -1.9 (-2.9 to -0.9) | | 0.77 (0.70-0.85) | <0.001 |
| Components of primary outcome |  |  |  |  | |  | |  |  |  | |  |  |
| Cardiovascular death | 59 | 5432 | 1.1 |  | | 82 | | 7266 | 1.1 | -0.0 (-4.1 to 0.3) | | 0.97 (0.76-1.24) | 0.804 |
| Ischaemic stroke | 134 | 5178 | 2.6 |  | | 268 | | 6777 | 4.0 | -1.4 (-2.0 to -0.7) | | 0.64 (0.55-0.74) | <0.001 |
| Hospitalisation for heart failure | 174 | 5133 | 3.4 |  | | 284 | | 6654 | 4.3 | -0.9 (-1.6 to -0.2) | | 0.78 (0.68-0.89) | <0.001 |
| Acute myocardial infarction | 17 | 5414 | 0.3 |  | | 41 | | 7195 | 0.6 | -0.3 (-0.5 to -0.0) | | 0.54 (0.35-0.82) | 0.004 |

Event rates are presented as per 100 person-years. CI, confidence interval; HFRS, Hospital Frailty Risk Score; HR, hazard ratio.

**Supplemental Table S10.** Outcomes in weighted patients undergoing rhythm or rate control in time-varying regression analyses which treatment was treated as a time-dependent variable.

|  | Number of events | Person-years | Event rate |  | Number of events | Person-years | Event rate | Weighted HR  (95% CI) | p value |
| --- | --- | --- | --- | --- | --- | --- | --- | --- | --- |
| ***Highly-frail (HFRS >15)*** | Rhythm control | | |  | Rate control | | |  |  |
| Primary composite outcome | 42 | 207 | 20.3 |  | 75 | 351 | 21.3 | 0.97 (0.74-1.26) | 0.799 |
| Components of primary outcome |  |  |  |  |  |  |  |  |  |
| Cardiovascular death | 12 | 241 | 5.0 |  | 38 | 446 | 8.6 | 0.57 (0.36-0.91) | 0.018 |
| Ischaemic stroke | 19 | 221 | 8.6 |  | 33 | 390 | 8.5 | 1.02 (0.68-1.53) | 0.937 |
| Hospitalisation for heart failure | 17 | 224 | 7.6 |  | 27 | 405 | 6.6 | 1.20 (0.77-1.85) | 0.421 |
| Acute myocardial infarction | 1 | 240 | 0.3 |  | 4 | 441 | 0.9 | 0.34 (0.05-2.32) | 0.271 |
| ***Moderately-frail (HFRS 5-15)*** | Rhythm control | | |  | Rate control | | |  |  |
| Primary composite outcome | 174 | 1456 | 11.9 |  | 329 | 2472 | 13.3 | 0.88 (0.77-1.00) | 0.068 |
| Components of primary outcome |  |  |  |  |  |  |  |  |  |
| Cardiovascular death | 43 | 1713 | 2.5 |  | 123 | 3033 | 4.1 | 0.60 (0.47-0.78) | <0.001 |
| Ischaemic stroke | 79 | 1588 | 5.0 |  | 147 | 2745 | 5.4 | 0.94 (0.77-1.15) | 0.550 |
| Hospitalisation for heart failure | 90 | 1558 | 5.8 |  | 154 | 2722 | 5.7 | 1.01 (0.84-1.22) | 0.905 |
| Acute myocardial infarction | 8 | 1688 | 0.5 |  | 16 | 3006 | 0.5 | 0.94 (0.52-1.69) | 0.835 |
| ***Non-frail (HFRS <5)*** | Rhythm control | | |  | Rate control | | |  |  |
| Primary composite outcome | 371 | 5341 | 7.0 |  | 785 | 9315 | 8.4 | 0.82 (0.75-0.90) | <0.001 |
| Components of primary outcome |  |  |  |  |  |  |  |  |  |
| Cardiovascular death | 85 | 6050 | 1.4 |  | 241 | 11125 | 2.2 | 0.64 (0.54-0.77) | <0.001 |
| Ischaemic stroke | 155 | 5695 | 2.7 |  | 376 | 10275 | 3.7 | 0.76 (0.66-0.87) | <0.001 |
| Hospitalisation for heart failure | 196 | 5642 | 3.5 |  | 395 | 10047 | 3.9 | 0.89 (0.78-1.01) | 0.068 |
| Acute myocardial infarction | 19 | 6014 | 0.3 |  | 51 | 11012 | 0.5 | 0.70 (0.48-1.04) | 0.075 |

Event rates are presented as per 100 person-years. CI, confidence interval; HFRS, Hospital Frailty Risk Score; HR, hazard ratio.

**Supplemental Table S11.** Outcomes in propensity score matched patients undergoing rhythm or rate control.

| **Outcome** | Number of events | Person-years | Event rate |  | | Number of events | | Person-years | Event rate | Absolute rate difference per 100 person-years  (95% CI) | | Weighted HR  (95% CI) | p value |
| --- | --- | --- | --- | --- | --- | --- | --- | --- | --- | --- | --- | --- | --- |
| ***Highly-frail (HFRS >15)*** | Rhythm control (N=514) | | |  | | Rate control (N=533) | | | |  | |  |  |
| Primary composite outcome | 83 | 424 | 19.6 |  | | 97 | | 427 | 22.7 | -3.1 (-9.3 to 3.0) | | 0.86 (0.64-1.14) | 0.290 |
| Components of primary outcome |  |  |  |  | |  | |  |  |  | |  |  |
| Cardiovascular death | 44 | 516 | 8.5 |  | | 49 | | 517 | 9.5 | -1.0 (-4.6 to 2.7) | | 0.91 (0.61-1.36) | 0.650 |
| Ischaemic stroke | 30 | 465 | 6.4 |  | | 37 | | 468 | 7.9 | -1.5 (-4.9 to 2.0) | | 0.83 (0.52-1.34) | 0.450 |
| Hospitalisation for heart failure | 29 | 471 | 6.2 |  | | 33 | | 469 | 7.0 | -0.9 (-4.2 to 2.4) | | 0.88 (0.54-1.44) | 0.610 |
| Acute myocardial infarction | 1 | 515 | 0.2 |  | | 4 | | 511 | 0.8 | -0.6 (-1.4 to 0.3) | | 0.25 (0.03-2.23) | 0.210 |
| ***Moderately-frail (HFRS 5-15)*** | Rhythm control (N=2500) | | | |  | | Rate control (N=2635) | | |  |  |  |  |
| Primary composite outcome | 384 | 3155 | 12.2 |  | | 431 | | 3157 | 13.7 | -1.5 (-3.3 to 0.3) | | 0.89 (0.78-1.02) | 0.096 |
| Components of primary outcome |  |  |  |  | |  | |  |  |  | |  |  |
| Cardiovascular death | 165 | 3764 | 4.4 |  | | 167 | | 3895 | 4.3 | 0.1 (-0.8 to 1.0) | | 1.03 (0.83-1.28) | 0.760 |
| Ischaemic stroke | 152 | 3467 | 4.4 |  | | 206 | | 3487 | 5.9 | -1.5 (-2.6 to -0.5) | | 0.74 (0.60-0.91) | 0.004 |
| Hospitalisation for heart failure | 184 | 3403 | 5.4 |  | | 178 | | 3516 | 5.1 | 0.3 (-0.7 to 1.4) | | 1.06 (0.86-1.30) | 0.590 |
| Acute myocardial infarction | 18 | 3735 | 0.5 |  | | 19 | | 3848 | 0.5 | -0.0 (-0.3 to 0.3) | | 0.98 (0.51-1.87) | 0.950 |
| ***Non-frail (HFRS <5)*** | Rhythm control (N=6520) | | | |  | | Rate control (N=7909) | | |  | | | |
| Primary composite outcome | 831 | 11240 | 7.4 |  | | 937 | | 10800 | 8.7 | -1.3 (-2.0 to -0.5) | | 0.87 (0.79-0.96) | 0.003 |
| Components of primary outcome |  |  |  |  | |  | |  |  |  | |  |  |
| Cardiovascular death | 311 | 13043 | 2.4 |  | | 330 | | 12938 | 2.6 | -0.2 (-0.5 to 0.2) | | 0.96 (0.82-1.12) | 0.600 |
| Ischaemic stroke | 337 | 12209 | 2.8 |  | | 451 | | 11909 | 3.8 | -1.0 (-1.5 to -0.6) | | 0.74 (0.64-0.85) | <0.001 |
| Hospitalisation for heart failure | 410 | 11961 | 3.4 |  | | 464 | | 11690 | 4.0 | -0.5 (-1.0 to -0.1) | | 0.89 (0.78-1.01) | 0.071 |
| Acute myocardial infarction | 38 | 12950 | 0.3 |  | | 69 | | 12788 | 0.5 | -0.2 (-0.4 to -0.1) | | 0.56 (0.38-0.83) | 0.004 |

Event rates are presented as per 100 person-years. CI, confidence interval; Hospital Frailty Risk Score; HR, hazard ratio.

**Supplemental Table S12.** Outcomes in weighted patients undergoing rhythm or rate control in whom performance of cardioversion within 180 days of their first record of prescription of rate control drugs was treated as intention-to-treat with a rhythm control strategy.

|  | **Rhythm control (n=9553*)** | | | **Rate control (n=11058*)** | | | **Absolute rate difference per 100 person-years***  **(95% CI)** | **Weighted hazard ratio (95% CI)** | **p value** |
| --- | --- | --- | --- | --- | --- | --- | --- | --- | --- |
| **Outcome** | **Number of events** | **Person**  **-years** | **Event rate** | **Number of events** | **Person -years** | **Event rate** |  |  |  |
| **Highly-frail (HFRS >15)** | n=177.2 | | | n=177.2 | | |  |  |  |
| Primary composite outcome | 64 | 317 | 20.4 | 71 | 326 | 21.6 | -1.2 (-8.3 to 5.9) | 0.93 (0.75-1.17) | 0.552 |
| Components of primary outcome |  |  |  |  |  |  |  |  |  |
| Cardiovascular death | 32 | 391 | 8.2 | 35 | 411 | 8.4 | -1.6 (-4.2 to 0.4) | 0.96 (0.69-1.33) | 0.805 |
| Ischaemic stroke | 27 | 348 | 7.7 | 30 | 365 | 8.1 | -0.4 (-4.5 to 3.7) | 0.93 (0.65-1.33) | 0.689 |
| Hospitalisation for heart failure | 23 | 357 | 6.5 | 23 | 376 | 6.1 | 0.4 (-3.3 to 4.0) | 1.03 (0.69-1.55) | 0.873 |
| Acute myocardial infarction | 1 | 391 | 0.2 | 4 | 398 | 1.0 | -0.8 (-1.9 to 0.3) | 0.22 (0.05-1.07) | 0.061 |
| Night spent in hospital/year† | 110.4 ± 129.7 | | | 112.2 ± 131.4 | | | -1.7 (-17.6 to 14.1) |  | 0.960 |
| **Moderately-frail (HFRS 5-15)** | n=851.1 | | | n=851.1 | | |  |  |  |
| Primary composite outcome | 273 | 2206 | 12.4 | 292 | 2142 | 13.7 | -1.3 (-3.4 to 0.8) | 0.92 (0.82-1.03) | 0.127 |
| Components of primary outcome |  |  |  |  |  |  |  |  |  |
| Cardiovascular death | 120 | 2633 | 4.6 | 112 | 2645 | 4.2 | 0.3 (-0.8 to 1.5) | 1.09 (0.91-1.30) | 0.342 |
| Ischaemic stroke | 110 | 2416 | 4.6 | 136 | 2384 | 5.7 | -1.2 (-2.4 to 0.1) | 0.80 (0.67-0.95) | 0.012 |
| Hospitalisation for heart failure | 122 | 2395 | 5.1 | 132 | 2365 | 5.6 | -0.5 (-1.8 to 0.8) | 0.92 (0.77-1.09) | 0.329 |
| Acute myocardial infarction | 12 | 2609 | 0.5 | 13 | 2614 | 0.5 | -0.0 (-0.4 to 0.3) | 0.92 (0.54-1.58) | 0.773 |
| Night spent in hospital/year† | 47.1 ± 87.8 | | | 52.6 ± 93.0. | | | -5.5 (-10.5 to -0.6) |  | 0.029 |
| **Non-frail (HFRS <5)** | n=2170.8 | | | n=2170.8 | | |  |  |  |
| Primary composite outcome | 584 | 8023 | 7,3 | 656 | 7606 | 8.6 | -1.3 (-2.2 to -0.5) | 0.86 (0.80-0.93) | <0.001 |
| Components of primary outcome |  |  |  |  |  |  |  |  |  |
| Cardiovascular death | 221 | 9309 | 2.4 | 235 | 9116 | 2.6 | -0.2 (-0.7 to 0.3) | 0.94 (0.82-1.07) | 0.329 |
| Ischaemic stroke | 243 | 8692 | 2.8 | 317 | 8360 | 3.8 | -1.0 (-1.5 to -0.5) | 0.75 (0.66-0.84) | <0.001 |
| Hospitalisation for heart failure | 285 | 8544 | 3.3 | 327 | 8272 | 4.0 | -0.6 (-1.2 to -0.0) | 0.86 (0.77-0.96) | 0.008 |
| Acute myocardial infarction | 27 | 9249 | 0.3 | 45 | 9006 | 0.5 | -0.2 (-0.4 to -0.0) | 0.60 (0.42-0.84) | 0.003 |
| Night spent in hospital/year† | 18.1 ± 44.8 | | | 21.7 ± 52.1 | | | -3.6 (-5.2 to -2.0) |  | <0.001 |

Event rates are presented as per 100 person-years. CI, confidence interval; HFRS, Hospital Frailty Risk Score. *Crude number of patients. †Results are reported as mean (standard deviation) and the difference between the treatment groups was estimated using a two-sample weighted t test.

**Supplemental Table S13.** Outcomes in weighted patients undergoing rhythm or rate control defined using a 30-day enrolment period after the first prescription instead of the 180-day period in the main analyses.

|  | **Rhythm control (N=11127*)** | | | **Rate control (N=12411*)** | | | **Absolute rate difference per 100 person-years***  **(95% CI)** | **Weighted hazard ratio (95% CI)** | **p value** |
| --- | --- | --- | --- | --- | --- | --- | --- | --- | --- |
| **Outcome** | **Number of events** | **Person**  **-years** | **Event rate** | **Number of events** | **Person -years** | **Event rate** |  |  |  |
| **Highly-frail (HFRS >15)** | n=283.0 | | | n=283.0 | | |  |  |  |
| Primary composite outcome | 181 | 499 | 36.2 | 193 | 475 | 40.6 | -4.4 (-12.2 to 3.3) | 0.91 (0.79-1.05) | 0.198 |
| Components of primary outcome |  |  |  |  |  |  |  |  |  |
| Cardiovascular death | 58 | 680 | 8.5 | 70 | 639 | 11.0 | -2.5 (-5.9 to 0.9) | 0.80 (0.63-1.02) | 0.070 |
| Ischaemic stroke | 58 | 563 | 10.3 | 70 | 531 | 13.2 | -2.9 (-7.0 to 1.2) | 0.81 (0.64-1.03) | 0.080 |
| Hospitalisation for heart failure | 45 | 591 | 7.6 | 41 | 577 | 7.1 | 0.4 (-2.7 to 3.5) | 1.09 (0.82-1.46) | 0.562 |
| Acute myocardial infarction | 6 | 666 | 0.9 | 8 | 620 | 1.3 | -0.4 (-1.5 to 0.8) | 0.74 (0.34-1.60) | 0.443 |
| Night spent in hospital/year† | 146.6 ± 145.9 | | | 147.0 ± 144.3 | | | -0.4 (-15.7 to 14.9) |  | 0.960 |
| **Moderately-frail (HFRS 5-15)** | n=1190.4 | | | n=1190.4 | | |  |  |  |
| Primary composite outcome | 569 | 3295 | 17.3 | 594 | 3133 | 19.0 | -1.7 (-3.8 to 0.4) | 0.92 (0.84-0.99) | 0.033 |
| Components of primary outcome |  |  |  |  |  |  |  |  |  |
| Cardiovascular death | 202 | 3931 | 5.1 | 187 | 3955 | 4,7 | 0.4 (-0.6 to 1.4) | 1.10 (0.96-1.26) | 0.170 |
| Ischaemic stroke | 193 | 3514 | 5.5 | 254 | 3360 | 7.6 | -2.1 (-3.3 to -0.9) | 0.73 (0.64-0.83) | <0.001 |
| Hospitalisation for heart failure | 194 | 3488 | 5.5 | 214 | 3474 | 6.2 | -0.6 (-1.8 to 0.5) | 0.90 (0.79-1.03) | 0.127 |
| Acute myocardial infarction | 24 | 3880 | 0.6 | 16 | 3914 | 0.4 | 0.2 (-0.1 to 0.5) | 1.45 (0.94-2.26) | 0.096 |
| Night spent in hospital/year† | 60.0 ± 100.9 | | | 64.8 ± 105.1 | | | -4.9 (-10.1 to -0.03) |  | 0.064 |
| **Non-frail (HFRS <5)** | n=2950.2 | | | n=2950.2 | | |  |  |  |
| Primary composite outcome | 1056 | 12279 | 8.6 | 1229 | 11528 | 1056 | 12279 | 8.6 | 1229 |
| Components of primary outcome |  |  |  |  |  |  |  |  |  |
| Cardiovascular death | 319 | 14079 | 2.3 | 354 | 13607 | 319 | 14079 | 2.3 | 354 |
| Ischaemic stroke | 389 | 13015 | 3.0 | 514 | 12277 | 389 | 13015 | 3.0 | 514 |
| Hospitalisation for heart failure | 441 | 12780 | 3.5 | 548 | 12044 | 441 | 12780 | 3.5 | 548 |
| Acute myocardial infarction | 48 | 13967 | 0.3 | 70 | 13424 | 48 | 13967 | 0.3 | 70 |
| Night spent in hospital/year† | 21.2 ± 51.5 | | | 25.5 ± 58.5 | | | -4.3 (-6.0 to -2.6) |  | <0.001 |

Event rates are presented as per 100 person-years. CI, confidence interval; HFRS, Hospital Frailty Risk Score. *Crude number of patients. †Results are reported as mean (standard deviation) and the difference between the treatment groups was estimated using a two-sample weighted t test.

**Supplemental Table S14.** Risk of 30 falsification endpoints in weighted patients undergoing rhythm control compared with rate control.

| **Endpoints** | **High-frail** | | **Intermediate-frail** | | **Non-frail** | |
| --- | --- | --- | --- | --- | --- | --- |
|  | **HR (95% CI)** | **P value** | **HR (95% CI)** | **P value** | **HR (95% CI)** | **p value** |
| Influenza | 0.79 (0.61-1.04) | 0.093 | 1.06 (0.70-1.62) | 0.778 | 0.56 (0.22-1.47) | 0.240 |
| Major fracture | 0.99 (0.86-1.14) | 0.871 | 1.17 (0.96-1.43) | 0.123 | 1.00 (0.67-1.49) | 0.989 |
| Urinary tract infection | 0.98 (0.91-1.06) | 0.574 | 1.04 (0.93-1.16) | 0.530 | 1.06 (0.85-1.32) | 0.605 |
| Tuberculosis | 0.97 (0.82-1.16) | 0.748 | 1.11 (0.71-1.15) | 0.408 | 0.83 (0.51-1.35) | 0.455 |
| Syphilis | 0.61 (0.37-1.01) | 0.056 | 0.65 (0.33-1.27) | 0.206 | 1.50 (0.35-6.38) | 0.581 |
| Viral enteritis | 1.09 (0.76-1.54) | 0.650 | 1.25 (0.67-2.35) | 0.488 | 0.43 (0.07-2.64) | 0.364 |
| Warts | 1.11 (0.62-1.97) | 0.724 | 1.63 (0.59-4.47) | 0.345 | 1.82 (0.25-12.99) | 0.552 |
| Stomach cancer | 0.93 (0.73-1.19) | 0.578 | 0.71 (0.48-1.06) | 0.093 | 0.90 (0.37-2.63) | 0.979 |
| Bone malignancy | 0.73 (0.51-1.05) | 0.093 | 1.02 (0.53-1.96) | 0.947 | 0.26 (0.03-2.12) | 0.207 |
| Lymphoma | 1.10 (0.56-2.19) | 0.780 | 0.47 (0.12-1.89) | 0.286 | 1.13 (0.09-13.86) | 0.926 |
| Lipoma | 1.18 (0.81-1.73) | 0.383 | 1.37 (0.62-3.00) | 0.438 | 4.07 (0.34-48.36) | 0.266 |
| Carpal tunnel syndrome | 1.03 (0.70-1.49) | 0.896 | 1.48 (0.73-2.98) | 0.277 | 1.79 (0.27-11.73) | 0.545 |
| Hordeolum / chalazion | 1.07 (0.88-1.30) | 0.515 | 0.97 (0.66-1.43) | 0.890 | 0.95 (0.32-2.83) | 0.932 |
| Pterygium | 0.96 (0.73-1.26) | 0.749 | 1.33 (0.79-2.25) | 0.281 | 1.27 (0.25-6.57) | 0.776 |
| Otitis media | 0.96 (0.84-1.10) | 0.587 | 1.17 (0.93-1.48) | 0.181 | 1.12 (0.61-2.05) | 0.709 |
| Meniere's disease | 1.16 (0.93-1.45) | 0.198 | 1.25 (0.86-1.80) | 0.240 | 1.47 (0.54-4.03) | 9 |
| Acute appendicitis | 1.23 (0.80-1.89) | 0.356 | 0.90 (0.48-1.71) | 0.755 | 1.00 (0.16-6.28) | 1.000 |
| Diverticulitis of intestine | 1.39 (0.90-2.14) | 0.140 | 1.97 (0.98-3.96) | 0.058 | 0.70 (0.17-2.95) | 0.629 |
| Cholecystitis | 0.81 (0.62-1.06) | 0.127 | 0.90 (0.56-1.44) | 0.658 | 0.82 (0.32-2.03) | 0.656 |
| Urticaria | 0.99 (0.91-1.08) | 0.882 | 1.05 (0.91-1.22) | 0.489 | 1.15 (0.79-1.67) | 0.468 |
| Ingrowing nail | 1.10 (0.82-1.49) | 0.515 | 1.73 (1.04-2.85) | 0.033 | 0.48 (0.15-1.55) | 0.221 |
| Seropositive rheumatoid arthritis | 0.81 (0.54-1.22) | 0.308 | 0.89 (0.49-1.62) | 0.704 | 1.57 (0.42-5.81) | 0.502 |
| Gout | 0.91 (0.80-1.04) | 0.174 | 0.95 (0.76-1.19) | 0.643 | 1.26 (0.69-2.31) | 0.452 |
| Frozen shoulder | 0.96 (0.86-1.07) | 0.430 | 1.09 (0.90-1.32) | 0.372 | 1.16 (0.73-1.85) | 0.539 |
| Osteomyelitis | 1.20 (0.61-2.35) | 0.600 | 0.57 (0.25-1.33) | 0.193 | 1.66 (0.32-8.64) | 0.548 |
| Nausea and vomiting | 1.07 (0.99-1.15) | 0.112 | 1.07 (0.95-1.21) | 0.240 | 0.85 (0.66-1.10) | 0.219 |
| Dysuria | 0.99 (0.86-1.15) | 0.939 | 1.07 (0.87-1.31) | 0.548 | 0.77 (0.53-1.14) | 0.190 |
| Voice disturbance | 1.17 (0.53-2.56) | 0.705 | 0.90 (0.20-4.13) | 0.895 | * | * |
| Seizure | 0.77 (0.60-1.01) | 0.055 | 0.83 (0.60-1.14) | 0.240 | 0.78 (0.46-1.33) | 0.364 |
| Anaphylaxis/Allergic reaction | 0.87 (0.62-1.20) | 0.389 | 1.13 (0.65-1.96) | 0.658 | 0.91 (0.22-3.77) | 0.895 |

CI, confidence interval; HR, hazard ratio. * No events both in the rhythm and rate-control groups.

**Supplemental Figure S1.** Distributions of the propensity score before and after overlap weighting in non- frail (A), Moderately-frail (B), and Highly-frail (C).

**A. Non-frail (Hospital Frailty Risk Score <5)**

**
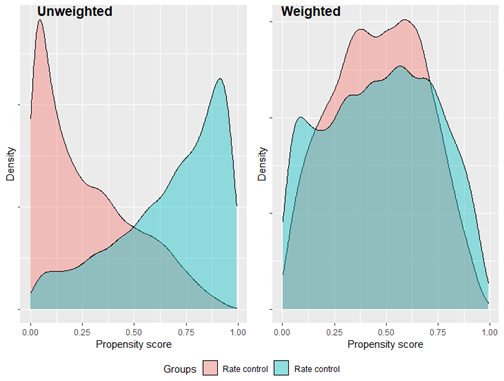
**

**B. Moderately-frail (Hospital Frailty Risk Score 5 - 15)**

**
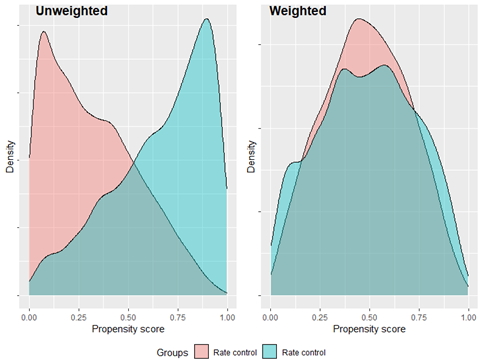
**

**C. Highly-frail (Hospital Frailty Risk Score >15)**

**
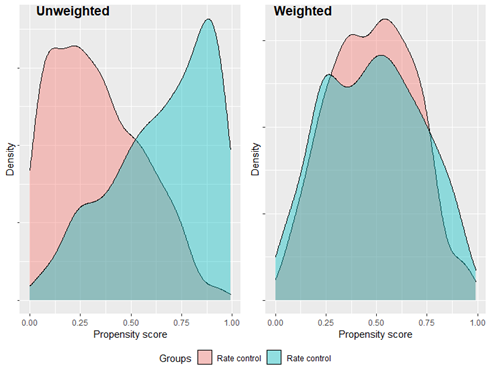
**

**Supplemental Figure S2.** Schematic diagram showing the analytical approach used in this study.


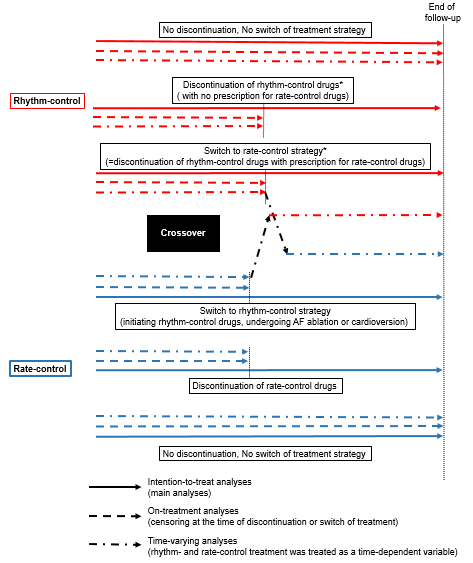


*No crossover was permitted in the rhythm-controlled patients who underwent ablation.

**Supplemental Figure S3.** Subgroup analyses of the primary composite outcome.


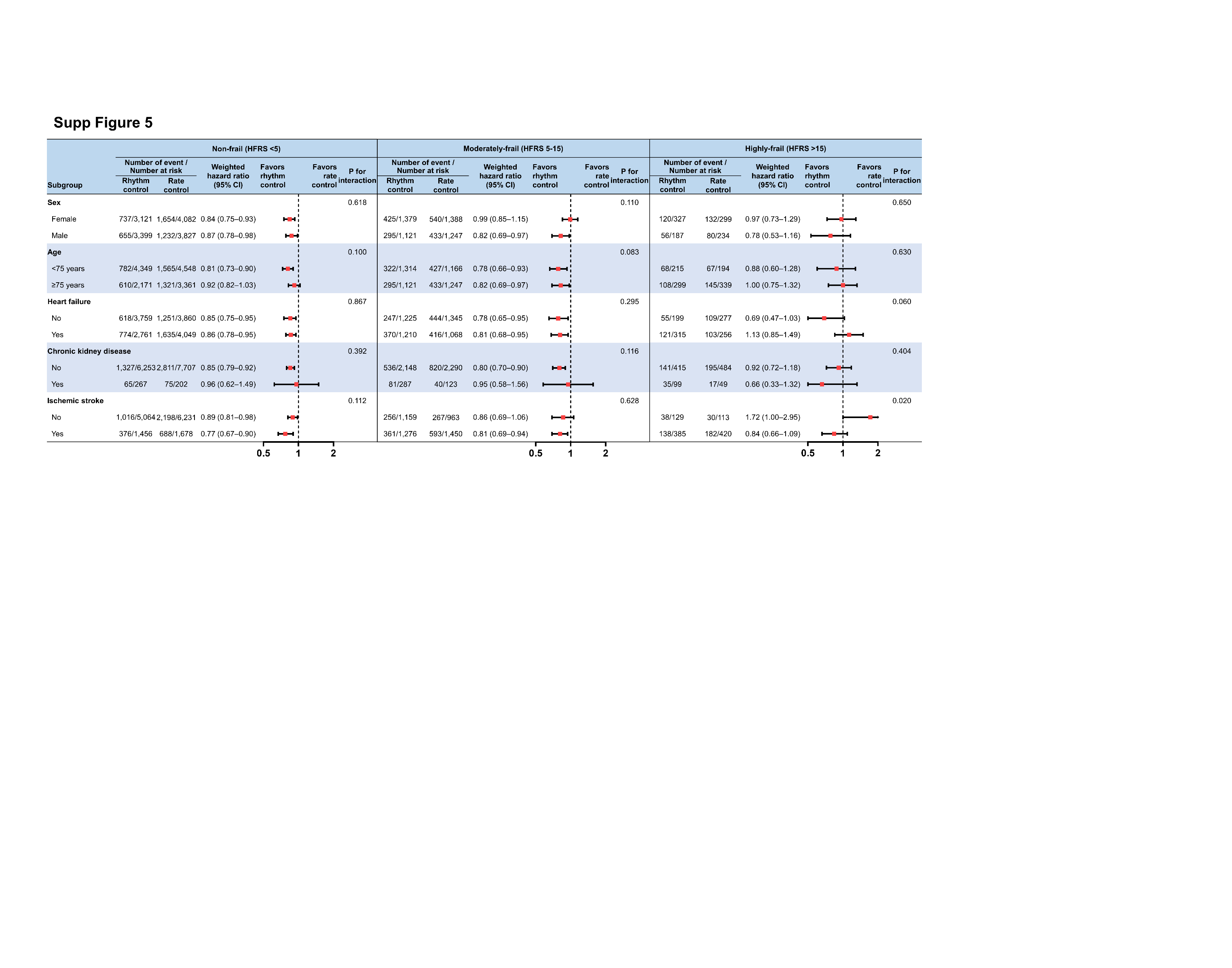


CI, confidence interval; Hospital Frailty Risk Score; HR, hazard ratio.
